# Deep amplicon sequencing for culture-free prediction of susceptibility or resistance to 13 anti-tuberculous drugs

**DOI:** 10.1183/13993003.02338-2020

**Published:** 2021-03-18

**Authors:** Agathe Jouet, Cyril Gaudin, Nelly Badalato, Caroline Allix-Béguec, Stéphanie Duthoy, Alice Ferré, Maren Diels, Yannick Laurent, Sandy Contreras, Silke Feuerriegel, Stefan Niemann, Emmanuel André, Michel K. Kaswa, Elisa Tagliani, Andrea Cabibbe, Vanessa Mathys, Daniela Cirillo, Bouke C. de Jong, Leen Rigouts, Philip Supply

**Affiliations:** 1 GenoScreen, Lille, France; 2 BCCM/ITM, Mycobacteria Collection, Institute of Tropical Medicine, Antwerp, Belgium; 3 Molecular and Experimental Mycobacteriology, Research Center Borstel, Borstel, Germany; 4 German Center for Infection Research (DZIF), Partner Site Hamburg–Lübeck–Borstel, Borstel, Germany; 5 Laboratory of Clinical Bacteriology and Mycology, Dept of Microbiology and Immunology, KU Leuven, Leuven, Belgium; 6 National Tuberculosis Program, Kinshasa, Democratic Republic of the Congo; 7 Emerging Bacterial Pathogens, IRCCS San Raffaele Scientific Institute, Milan, Italy; 8 Unit Bacterial Diseases Service, Infectious Diseases in Humans, Sciensano, Brussels, Belgium; 9 Mycobacteriology Unit, Dept of Biomedical Sciences, Institute of Tropical Medicine, Antwerp, Belgium; 10 Dept of Biomedical Sciences, Antwerp University, Antwerp, Belgium; 11 Université de Lille, CNRS, INSERM, CHU Lille, Institut Pasteur de Lille, U1019–UMR 8204–CIIL (Center for Infection and Immunity of Lille), Lille, France; 12 These authors contributed equally to this work

## Abstract

Conventional molecular tests for detecting *Mycobacterium tuberculosis* complex (MTBC) drug resistance on clinical samples cover a limited set of mutations. Whole-genome sequencing (WGS) typically requires culture.

Here, we evaluated the Deeplex Myc-TB targeted deep-sequencing assay for prediction of resistance to 13 anti-tuberculous drugs/drug classes, directly applicable on sputum.

With MTBC DNA tests, the limit of detection was 100–1000 genome copies for fixed resistance mutations. Deeplex Myc-TB captured *in silico* 97.1–99.3% of resistance phenotypes correctly predicted by WGS from 3651 MTBC genomes. On 429 isolates, the assay predicted 92.2% of 2369 first- and second-line phenotypes, with a sensitivity of 95.3% and a specificity of 97.4%. 56 out of 69 (81.2%) residual discrepancies with phenotypic results involved pyrazinamide, ethambutol and ethionamide, and low-level rifampicin or isoniazid resistance mutations, all notoriously prone to phenotypic testing variability. Only two out of 91 (2.2%) resistance phenotypes undetected by Deeplex Myc-TB had known resistance-associated mutations by WGS analysis outside Deeplex Myc-TB targets. Phenotype predictions from Deeplex Myc-TB analysis directly on 109 sputa from a Djibouti survey matched those of MTBSeq/PhyResSE/Mykrobe, fed with WGS data from subsequent cultures, with a sensitivity of 93.5/98.5/93.1% and a specificity of 98.5/97.2/95.3%, respectively. Most residual discordances involved gene deletions/indels and 3–12% heteroresistant calls undetected by WGS analysis or natural pyrazinamide resistance of globally rare “*Mycobacterium canettii*” strains then unreported by Deeplex Myc-TB. On 1494 arduous sputa from a Democratic Republic of the Congo survey, 14 902 out of 19 422 (76.7%) possible susceptible or resistance phenotypes could be predicted culture-free.

Deeplex Myc-TB may enable fast, tailored tuberculosis treatment.

## Abstract 2

**The novel Deeplex Myc-TB molecular assay shows a high degree of accuracy for extensive prediction of susceptibility and resistance to 13 anti-tuberculous drugs, directly achievable without culture, which may enable fast, tailored tuberculosis treatment** https://bit.ly/3bAvcAt

## Introduction

Important gaps remain for the diagnosis of drug-resistant tuberculosis (TB). Less than a third of the around 484 000 multidrug (MDR)- or rifampicin-resistant TB incident cases estimated in 2018 were diagnosed and treated [1]. Phenotypic drug susceptibility testing (pDST) takes weeks of culture, while conventional molecular tests only identify common drug resistance mutations in a few gene targets [2].

Whole-genome sequencing (WGS) can effectively predict drug resistance or susceptibility of *Mycobacterium tuberculosis* complex (MTBC) strains [3–5]. However, its routine use for mycobacterial diagnosis typically requires primary culture [6]. Even if complex DNA enrichment procedures or thermoprotective DNA extraction are used, sequence coverage depths obtained by WGS directly on specimens often remain poor, even on samples with high bacterial loads [7, 8]. This limits the sensitivity and/or the degree of confidence for detecting resistance mutations, especially when borne by minority populations (defining heteroresistance) that can be predictive of treatment failure [9].

Targeted, amplicon-based deep sequencing represents an attractive alternative [10]. Selective amplification of relevant gene regions prior to sequencing reduces both the amount of required DNA and the interference of unrelated DNA sequences (human or from microbial flora). Sequence coverage depths and multiplexing of samples per sequencing run can be substantially increased [11].

A previously described amplicon-based assay targets predefined high-confidence resistance variants in six *M. tuberculosis* genomic regions only [11, 12]. In contrast, the targeted deep-sequencing assay called Deeplex Myc-TB (GenoScreen, Lille, France) targets full sequences (*i.e.* coding sequence plus part of promoter region) or (most) relevant regions of 18 MTBC drug resistance-associated genes, combined with genomic targets for mycobacterial species identification and MTBC strain genotyping. Included in the assay, a fully automated web application is used for rapid and user-friendly analysis and interpretation of the sequencing data, obtained from Illumina sequencers. Variant detection is comprehensive, including not only mutations known to be associated with resistance or susceptibility, but also as-yet uncharacterised mutations that can be confronted with drug susceptibility phenotypes when available. This assay has already been used in national and regional TB drug resistance surveys, including those under the supervision of the World Health Organization (WHO) [13–15], prior to its extensive evaluation.

Here, we describe for the first time the details of the assay and evaluate its performance based on data from more than 4000 isolates and 1600 clinical specimens.

## Methods

### Limit of detection

The limit of detection of Deeplex Myc-TB was evaluated using purified DNA from well-characterised MTBC strains of the WHO Special Programme for Research and Training in Tropical Diseases (TDR) collection (now included in the Belgian Culture Collection of Microorganisms (BCCM), Antwerp, Belgium) [16]. Serial dilutions were done after DNA quantification using the Qubit dsDNA HS Assay (Thermo Fisher, Waltham, MA, USA). A *Mycobacterium intracellulare* strain from BCCM was used for evaluation of the limit of detection for nontuberculous mycobacteria (NTM) identification. After Deeplex Myc-TB amplification as per manual instructions, amplicon libraries were prepared using the Nextera XT kit and sequenced with 150-bp paired-end reads using a MiSeq sequencer (Illumina, San Diego, CA, USA). For the subsequent stages, analyses were performed automatically using the integrated bioinformatics pipeline version 1.3 implemented in the Deeplex Myc-TB web application (note S1 in the supplementary material).

### *In silico* analysis of drug resistance prediction *versus* WGS

The catalogue of resistance-associated variants included in Deeplex Myc-TB was compared with the resistance determinants and their associated resistance phenotypes found in both the training set of 2099 MTBC genomes and those retrieved in the validation set of 1552 MTBC genomes published in Walker *et al.* [3].

### Deeplex Myc-TB phenotype prediction *versus* WGS and phenotypic testing

Comparisons of Deeplex Myc-TB variant detection and phenotype predictions *versus* WGS and pDST were performed using a set of 429 reference isolates, including 213 collected by the Belgian National TB Reference Center (Sciensano) between 2007 and 2015 (MDR strains) and between March and October 2013 (non-MDR strains) and 216 from the WHO-TDR collection [16]. For the WHO-TDR collection, pDST was performed by using the proportion method (on Löwenstein–Jensen or Middlebrook 7H11 agar medium for first- or second-line drugs, respectively) with critical concentrations of 0.2, 40, 4, 2, 2, 6, 10 and 10 μg·mL^−1^ isoniazid, rifampicin, streptomycin, ethambutol, ofloxacin, kanamycin, capreomycin and ethionamide, respectively, as described by Vincent *et al.* [16]. For the Sciensano strain set, pDST was routinely done using the BACTEC MGIT960 system and TB-eXiST application (Becton Dickinson, Franklin Lakes, NJ, USA) for first-line drugs as per the manufacturer's instructions, and the radiometric BACTEC 460 TB system (Becton Dickinson) for second-line drugs including amikacin, ofloxacin, moxifloxacin, ethionamide and linezolid, as described in Pfyffer *et al.* [17] or Cambau *et al.* [18] for isolates before or after 2012, respectively. Deeplex Myc-TB testing was used as described earlier. WGS was performed at GenoScreen using the Nextera XT kit and 150-bp paired-end reads on a HiSeq 2500 sequencer (Illumina) in Rapid Run mode. WGS analysis was performed using a Bowtie2-based pipeline with an initial threshold of 85% allele frequency for variant calling, with subsequent search for minority variants under low-frequency detection mode, using at least one read in both forward and reverse directions, 5× read coverage, and a minimal Phred score of 30 to call an allele with a minimal frequency of 3%. To avoid probable errors due to mislabelling of samples, Deeplex Myc-TB- and WGS-based phylogenetic lineage and spoligotype identifications were compared for consistency, and any isolate with more than three discordances between predicted and observed phenotypes was excluded from the analysis, as done elsewhere [3].

### Deeplex Myc-TB on clinical specimens

Deeplex Myc-TB sequencing data from 109 sputum samples and WGS data from cultured isolates from the Djibouti survey were obtained as described in Tagliani *et al.* [13]. Phenotype prediction from WGS data was performed by MTBSeq version 1.0.2 [19], PhyResSE version 1.0 with resistance-associated variant database version 29 [20] and Mykrobe version 0.8.1 [21].

Deeplex Myc-TB sequencing data of 1494 sputum samples from the Democratic Republic of the Congo (DRC) survey were obtained as described in Kayomo *et al.* [22]. Briefly, after Ziehl–Neelsen staining and standard smear microscopy grading of acid-fast bacilli (AFB), sputa were stored and transported in 96% ethanol at a ratio of 1:1 at room temperature. DNA was extracted using a Maxwell 16 Low Elution Volume DNA Purification System (Promega, Madison, WI, USA), and analysed with Deeplex Myc-TB kits using NextSeq and MiSeq platforms.

### Data availability

Deeplex Myc-TB sequence reads were deposited in the National Center for Biotechnology Information Sequence Read Archive (SRA) under BioProject numbers PRJNA649788 (TDR/Sciensano), PRJNA633444 (Djibouti), PRJNA643157 and PRJNA643242 (DRC), PRJNA633380 (NTM), and PRJNA633106 (limit of detection analysis). WGS SRAs are available under BioProject numbers PRJEB31023 for the TDR collection, PRJNA393924 for the Djibouti dataset and PRJEB25999 for the Sciensano collection. A detailed description of datasets used in this study is available in supplementary table S1.

## Results

### Assay design, limit of detection and mycobacterial species identification

**FIGURE 1:**
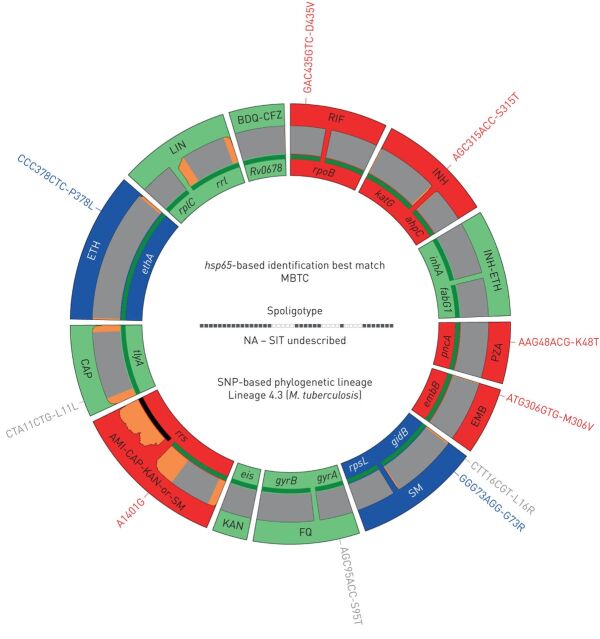
Deeplex Myc-TB results identifying a pre-extensively drug-resistant *Mycobacterium tuberculosis* complex (MTBC) strain in a sputum DNA sample collected in a tuberculosis drug resistance survey conducted in the Democratic Republic of the Congo. RIF: rifampicin; INH: isoniazid; PZA: pyrazinamide; EMB: ethambutol; SM: streptomycin; FQ: fluoroquinolones; KAN: kanamycin; AMI: amikacin; CAP: capreomycin; ETH: ethionamide; LIN: linezolid; BDQ: bedaquiline; CFZ: clofazimine; NA: not applicable; SIT: spoligotype international type; SNP: single nucleotide polymorphism; LOD: limit of detection. Information on *hsp65* best-match-based identification, spoligotype (in this case, not yet known to the SITVIT database) and phylogenetic SNP-based identification of MTBC lineage is shown in the centre of circle. Information on drug susceptibility and drug resistance predictions for 13 anti-tuberculous drugs/drug classes is as follows. Target gene regions are grouped within sectors in a circular map according to the anti-tuberculous drug resistance with which they are associated. Sectors in red and green indicate targets in which resistance-associated mutations or either no mutation or only mutations not associated with resistance (shown in grey) are detected, resulting in predictions of resistant or susceptible phenotypes, respectively. Blue sectors refer to regions where as-yet uncharacterised mutations are detected. Green lines above/below gene names represent the reference sequences with coverage breadth >95%. LOD of heteroresistance (reflected by subpopulations of reads bearing a mutation) depends on the read depth at mutation position and is shown either as grey (LOD 3%) or orange zones (LOD >3–80%) above/below reference sequences. Here, LOD is >3% at the end of a few targets only and over two *rrs* regions with usual lower coverage.

The major gene targets associated with resistance to 13 first- and second-line anti-tuberculous drugs/drug classes and the databases implemented in the web application are shown in figure 1, and tables 1 and 2. Further details on the assay design are provided in note S2 in the supplementary material, including the additional targets amplified in the single 24-plex PCR for mycobacterial species identification, spoligotyping/single nucleotide polymorphism (SNP) typing and an internal control, and the analysis with the web application.

**TABLE 1:** Mycobacterial/*Mycobacterium tuberculosis* complex (MTBC) genes or gene regions targeted by Deeplex Myc-TB

**Target**	**Genome position**	**Gene position**	**Codons**
***rpoB1***	760 957–761 355	1151–1549	384–517
***rpoB2***	760 280–760 812	474–1006	158–336
***inhA***	1 674 287–1 674 880	86–679	29–227
***fabG1***	1 673 321–1 673 755	−119–316	NA–106
***katG***	2 155 858–2 155 140	254–972	85–324
***ahpC***	2 726 030–2 726 585	−163–393	NA–131
***pncA***	2 289 301–2 288 672	−60– +9	Full CDS
***embB***	4 247 376–4 248 065	863–1552	288–518
***gidB***	4 408 185–4 407 411	18– +117	6–NA
***rpsL***	781 536–781 979	−24– +45	Full CDS
***rrs1***	1 472 561–1 473 417	716–1572	NA
***rrs2***	1 471 848–1 472 524	3–679	NA
***eis***	2 715 528–2 715 171	−196–162	NA–54
***tlyA***	1 917 811–1 918 750	−129– +4	Full CDS
***gyrA***	7377–7754	76–453	26–151
***gyrB***	6298–6943	1059–1704	353–568
***ethA***	4 327 482–4 325 951	−9– +53	Full CDS
***rplC***	801 108–801 483	300– +21	100–NA
***rrl***	1 475 923–1 476 625	2266–2968	NA
***Rv0678***	778 976–779 539	−14– +52	Full CDS
***hsp65***	528 772–529 172	165–565	55–189

CDS: coding sequence; NA: not applicable for codons (positions outside a CDS, or in *rrs* or *rrl* rDNA regions). Positions of the reference sequences relative to the genome and genes of the *M. tuberculosis* H37Rv strain are indicated. Genome positions are indicated according to forward or reverse orientations of corresponding genes. For gene positions, minus (–) or plus (+) signs indicate positions in promoter or 3′ regions relative to the +1 or last nucleotide of CDSs, respectively.

**TABLE 2:** Databases implemented in the Deeplex Myc-TB web application for mycobacterial species identification, *Mycobacterium tuberculosis* complex (MTBC) genotyping, and drug susceptibility and drug resistance prediction

**Database use**	**Database name**	**Reference**
**Species identification**	*hsp65*	[23]
**Spoligotyping**	SITVITWEB	[24]
**Lineage identification**	Coll	[25]
	PhyResSE	[20]
	Walker	[3]
**Drug resistance prediction^#^**	Miotto	[26]
	PhyResSE	[20]
	Walker	[3]
	ReSeqTB	[27]

^#^: priority is given to the collaborative, curated database ReSeqTB when detected variants are known to this database.

**FIGURE 2:**
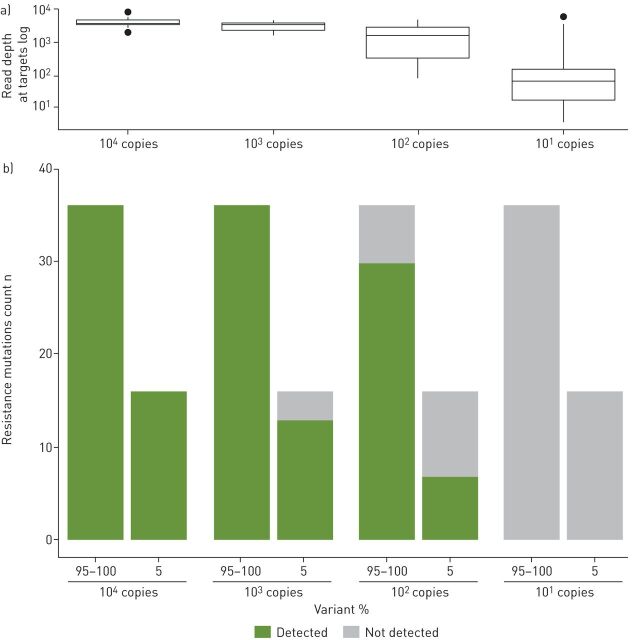
Limit of detection (LOD) of Deeplex Myc-TB for resistance variant detection. a) Read depth at resistance-associated Deeplex Myc-TB targets *versus* the number of input genomes. Box and whisker plots show median with interquartile range (IQR) and minimum–maximum range with a maximum of 1.5 IQR, respectively; outliers are indicated. b) For each dilution level with 10^1^, 10^2^, 10^3^ and 10^4^ genome copies, LOD was measured as the fraction of detected or undetected resistance variants in total sets of 36 (near-)fixed (95–100% frequency) and 16 minority (5% frequency) mutations, spread across four independent replicated tests of four different *Mycobacterium tuberculosis* complex genomic DNA extracts.

As Deeplex Myc-TB does not depend on a specific DNA extraction method, the assay's limit of detection was estimated as the fraction of detectable sequence variants in four replicated analyses using serially diluted purified, pre-quantified genomic DNA from three well-characterised MTBC strains and a mixture of two strains at a 5:95% ratio. All (near-)fixed resistance variants were detectable with 10^4^ and 10^3^ genomes, and 83.3% with 10^2^ genomes (complete variant profiles obtained for 13 out of 16 tests) (figure 2). For resistance variants at 5% frequency, these fractions were 100%, 81.3% (with complete minor variant profiles in three out of four tests) and 43.8% (none with a complete minor variant profile) with 10^4^, 10^3^ and 10^2^ genomes, respectively. Fixed and minority variants were not detected with 10^1^ genomes only (for limit of detection for MTBC and NTM identification, see supplementary figure S1 and S2, and note S1 in the supplementary material).

Out of 370 strains from 73 different NTM species/species complexes tested using DNA extracted from culture, 292 strains were identified at (sub)species or species complex levels by both reference and Deeplex Myc-TB testing. Of these, 274 (93.5%) were correctly identified at (sub)species or species complex level by Deeplex Myc-TB (supplementary figure S3 and supplementary table S2, and note S7 in the supplementary material for methods and details). The 18 (6.2%) strains that had taxonomically discordant results even at the complex level between both methods mostly consist of single discordant cases among otherwise partially/fully concordant strains for a species (*e.g. Mycobacterium ulcerans* (n=1/13) and *Mycobacterium kansasii* (n=1/14)) or species rarely involved in infections (*e.g. Mycobacterium peregrinum* (n=2)). However, for some of these few individual isolates of otherwise well-identified species, the correctness of the Deeplex Myc-TB identification was actually often possible or probable, as conflicting or ambiguous identifications were seen between the *rpoB* and 16S rDNA reference probes, with one or the other partially or fully matching the Deeplex Myc-TB result (*e.g.* *M. kansasii versus Mycobacterium gastri*) (supplementary table S2). The same often held true for the 16 residual isolates with discrepant subspecies within a matching complex (*e.g. M. intracellulare versus Mycobacterium chimaera)*.

### Resistance variant detection *versus* WGS *in silico*

We evaluated *in silico* the ability of Deeplex Myc-TB to capture 120 anti-tuberculous drug resistance-determining mutations spread across 14 genes, along with their concurrent first- and second-line resistance phenotypes, algorithmically identified in a WGS study by Walker *et al.* [3]. Of these 120 resistance determinants, 106 (88.3%) are included in the Deeplex Myc-TB targets and variant catalogue (supplementary table S3), spread across 13 of the 14 genes, the exception being *rpsA*, a minor target associated with pyrazinamide resistance. With these 106 variants, 644 out of the 663 (97.1%) resistant phenotypes predicted by WGS in the Walker *et al.* [3] training set of 2099 MTBC isolates were retrieved. Likewise, 53 out of the 58 resistance-determining mutations from the training set that were retrieved in the Walker *et al.* [3] validation set of 1552 isolates were captured by Deeplex Myc-TB, enabling the prediction of 1199 out of 1207 (99.3%) concurrent resistant phenotypes in this dataset (supplementary table S4).

### Phenotype prediction on isolates *versus* WGS and phenotypic testing

We compared the ability of both Deeplex Myc-TB and Illumina-based WGS analysis to detect variants in DNA extracts from 429 MTBC strains. Of the 2403 variants identified in the Deeplex Myc-TB targets by either method, 2373 (98.8%; 2293 SNPs and all 80 indels), including 797 (99.9%) resistance variants, were detected by both Deeplex Myc-TB and our WGS pipeline under low-frequency mode (validated for accurate SNP calling in accordance with recent guidelines [28]). The remaining 30 (1.2%) SNPs were all minority variants mostly with frequencies of ∼3–10% only identified by targeted deep sequencing, including one mutation associated with resistance (to ethambutol; note S4 in the supplementary material and supplementary table S5).

Deeplex Myc-TB drug susceptibility predictions based on these 2403 variants were compared with pDST results. In this set, 268 isolates were phenotypically resistant to at least one drug, including 156 MDR and six extensively drug-resistant isolates, resulting in 664 resistant and 1705 susceptible phenotypes. Of these 2369 phenotypes, 2184 (92.2%) were predicted by Deeplex Myc-TB with a mean sensitivity of 95.3% and a mean specificity of 97.4% (table 3 and supplementary table S6). The remaining 185 phenotypes (7.8%) could not be predicted due to the presence of mutations uncharacterised in the variant database. When results were stratified by type of phenotypic method used as a reference, the concordance with genotypic predictions was slightly higher for phenotypes tested by liquid culture (supplementary table S7) compared with those tested by solid culture (supplementary table S8), for the three drugs principally assayed with both types of methods (rifampicin, isoniazid and ethambutol).

**TABLE 3:** Deeplex Myc-TB phenotype predictions *versus* phenotypic drug susceptibility testing (pDST) on 429 reference isolates from the World Health Organization TDR and the Belgian National TB Reference Center (Sciensano) collections

	**Phenotypically resistant**					**Phenotypically susceptible**					**All**		**Excluding uncharacterised**		**Uncharacterised** **%**
	**Deeplex Myc-TB n**				**Total n**	**Deeplex Myc-TB n**				**Total n**	**Sensitivity** **%**	**Specificity** **%**	**Sensitivity** **%**	**Specificity** **%**	
	**R**	**R_h_**	**S**	**U**		**R**	**R_h_**	**S**	**U**						
**Rifampicin**	159	0	1	2	162	3	0	253	6	262	98.1 (94.7–99.4)	98.9 (96.7–98.2)	99.4 (96.5–99.9)	98.8 (96.6–99.6)	1.9
**Isoniazid^#^**	176	0	3	8	187	3	0	200	34	237	94.1 (89.8–96.7)	98.7 (96.3–99.6)	98.3 (95.2–99.4)	98.5 (95.7–99.5)	9.9
**Pyrazinamide^¶^**	39	3	7	4	53	0	0	146	5	151	79.2 (66.5–88.0)	100 (97.5–100)	85.7 (73.3–92.9)	100 (97.4–100)	4.4
**Ethambutol**	95	0	8	5	108	26	2	285	3	316	88.0 (80.5–92.8)	91.1 (87.5–93.8)	92.2 (85.4–96.0)	91.1 (87.4–93.7)	1.9
**Streptomycin**	49	0	5	36	90	1	0	91	33	125	54.4 (44.2–64.3)	99.2 (95.6–99.9)	90.7 (80.1–96.0)	98.9 (94.1–99.8)	32.1
**Fluoroquinolones^+^**	17	1	1	2	21	0	0	183	13	196	85.7 (65.4–95.0)	100 (98.1–100)	94.7 (75.4–99.1)	100 (97.9–100)	6.9
**Amikacin**	9	0	0	0	9	0	0	184	14	198	100 (70.1–100)	100 (98.1–100)	100 (70.1–100)	100 (97.9–100)	6.8
**Kanamycin**	2	1	1	0	4	0	0	3	0	3	75.0 (30.1–95.4)	100 (43.9–100)	75.0 (30.1–95.4)	100 (15.0–85.0)	0
**Capreomycin**	2	1	1	0	4	0	0	5	0	5	75.0 (30.1–95.4)	100 (56.6–100)	75.0 (30.1–95.4)	100 (15.0–85.0)	0
**Ethionamide**	18	1	1	6	26	6	0	172	12	190	73.1 (53.9–86.3)	96.8 (93.3–98.5)	95.0 (76.4–99.1)	96.6 (92.8–98.4)	8.3
**Linezolid**	0	0	0	0	0	0	0	20	2	22	NA	100 (85.1–100)	NA	100 (83.8–100)	9.1
**Total**	566	7	28	63	664	39	2	1542	122	1705	86.3 (83.5–88.7)	97.6 (96.7–98.2)	95.3 (93.3–96.8)	97.4 (96.5–98.1)	7.8

Data for sensitivity and specificity are presented as mean (95% CI); total sensitivity and specificity data are weighted means. Deeplex Myc-TB predictions were compared with phenotypes separately for each drug, across isolates with this data available. The unit of analysis was therefore a phenotype, not an isolate. Results for bedaquiline/clofazimine are not shown, as there was no pDST comparator. R: detection of at least one fixed resistance-associated mutation; R_h_: detection of resistance due to a minority variant (heteroresistance); S: detection of mutations known not to be associated with resistance (phylogenetic, benign, synonymous) or no mutation detected; U: detection of at least one nonsynonymous uncharacterised mutation in the absence of a resistance-associated mutation; NA: not applicable; WGS: whole-genome sequencing. ^#^: a beta version of the Deeplex Myc-TB kit used for this analysis did not yet include the *ahpC* promoter region including two isoniazid resistance-associated mutations G-48A and C-57T (note S2 in the supplementary material). We checked for these two mutations in the WGS dataset and found only two samples (TB-TDR0013 and TB-TDR-0160) with a fixed G-48A mutation. They were considered in this comparison to reflect results as obtained with the up-to-date version containing this region, as Deeplex Myc-TB captured 100% of the variants detected by WGS in the initial *ahpC* gene part and all other gene targets in this dataset (see text). ^¶^: a simulation of the inclusion of additional pyrazinamide resistance-associated mutations published in Yadon *et al.* [29] (not incorporated in the ReSeqTB and Deeplex Myc-TB databases) resulted in identification of only three additional resistance mutations, classified as uncharacterised by Deeplex Myc-TB. However, out of these three mutations, two were found in pyrazinamide-susceptible phenotypes and only one in a pyrazinamide-resistant phenotype. ^+^: fluoroquinolones include ofloxacin and moxifloxacin.

The proportion of resistant phenotypes accurately predicted as resistant by Deeplex Myc-TB was >90% (90.7% for streptomycin to 100% for amikacin) for most individual drugs, except for pyrazinamide (85.7%), kanamycin and capreomycin (75.0%, but reflecting four isolates only in both cases) (table 3). For the MDR-defining compounds rifampicin and isoniazid, 159 out of 160 (99.4%) and 176 out of 179 (98.3%) isolates with resistance variants were correctly classified as resistant, accounting for 98.1% and 94.1% of all rifampicin- and isoniazid-resistant isolates, respectively. For pyrazinamide, although all (42 out of 42 (100%)) isolates containing resistance variants for this drug were correctly predicted as resistant, these accounted for only 79.2% of all isolates with phenotypic resistance to pyrazinamide. Of note, seven minority resistance variants (with frequencies of 21.1–77%) successfully predicted resistance to pyrazinamide (n=3), fluoroquinolones, kanamycin, capreomycin and ethionamide (n=1 each).

Of the 601 resistant phenotypes with Deeplex Myc-TB predictions (*i.e.* not uncharacterised), only 28 (4.7%) were predicted as susceptible due the absence of resistance-associated mutation in the Deeplex Myc-TB targets. In these phenotypes, the possible presence of high-confidence resistance-associated mutations was searched in extended regions (full coding sequence and promoter regions) of genes from which only (most) critical regions are covered by Deeplex Myc-TB, as well in 14 other, secondary resistance-associated gene targets, outside the Deeplex Myc-TB target regions, such as the *embA* promoter region (including, *e.g.* the C-12T and C-16T mutations) or the extended *ethA* promoter region (including, *e.g.* the T-11C mutation) (supplementary table S9). WGS analysis detected a high-confidence resistance mutation in these regions for only one of these phenotypes (ethionamide resistant; L203L in *fabG1* [4]) (supplementary table S9). Likewise, *fabG1* L203L was the sole established resistance-associated mutation (for an isoniazid-resistant phenotype) detected by WGS outside of the assay's targets in 63 phenotypes uncharacterised by Deeplex Myc-TB.

Of the 1583 susceptible phenotypes with prediction, only 41 (2.6%) were discordantly predicted as resistant. They all involved ethambutol and *embB* mutations, ethionamide and *ethA* frameshift-causing indels (mechanistically expected to cause ethionamide resistance [30]), or known low-level isoniazid (*inhA* S94A, *ahpC* G-48A), rifampicin (*rpoB* L452P, H445N and D435Y) or streptomycin (*gidB* A138V) resistance mutations, all notoriously associated with poor phenotypic reproducibility (note S4 in the supplementary material) [3, 27, 31–33].

As acquisition of resistance to isoniazid is generally the first step towards drug resistance [34], a predicted susceptibility to isoniazid could *a contrario* be considered to predict susceptibility to other first-line drugs, for which gene targets have no drug resistance mutations but contain uncharacterised mutations, as shown for WGS-based analysis [4]. When doing so for Deeplex Myc-TB predictions, the diagnostic performance could be further improved, with proportions of uncharacterised phenotypes reduced to 0.5–2.5% for rifampicin, ethambutol and pyrazinamide, at the cost of a single incorrect prediction of a susceptible phenotype for each of these drugs (note S5 in the supplementary material and supplementary table S10). This conditional interpretation is left to the user's decision and not implemented in the Deeplex Myc-TB analysis algorithm.

### Deeplex Myc-TB on clinical specimens

We compared variant detection and phenotype predictions based on available Deeplex Myc-TB sequencing data directly obtained from 109 clinical specimens from a nationwide survey conducted in Djibouti *versus* analysis of WGS data obtained after culturing [13]. Overall, 693 out of 752 total variants (92.2%) were detected by both Deeplex Myc-TB and our WGS pipeline used under low-frequency detection mode. Of 59 (7.8%) discordances, all but one (a fixed *pncA* SNP not detected by Deeplex Myc-TB, albeit on a well-covered position) were minority variants undetected by this WGS pipeline, consistent with the average coverage depth of 3921× at Deeplex targets by Deeplex Myc-TB *versus* 57× by WGS (note S6 in the supplementary material and supplementary table S11).

Deeplex Myc-TB phenotype predictions were matched to those obtained with WGS data independently analysed with the WGS-based analysis tools PhyResSE [20], Mykrobe [21] and MTBSeq [19]. In contrast to PhyResSE and Mykrobe, MTBSeq requires bioinformatic skills for local implementation and use. However, MTBSeq has a more complete resistance mutation panel [19] and was therefore used as a primary reference in the comparison.

**FIGURE 3:**
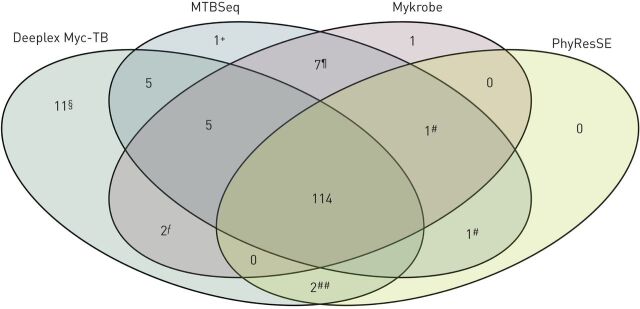
Venn diagram representing the agreement between resistant phenotypes identified by four *Mycobacterium tuberculosis* resistance and susceptibility prediction tools: Deeplex Myc-TB, MTBSeq, Mykrobe and PhyResSE. WGS: whole-genome sequencing. The numbers of resistant phenotypes predicted by Deeplex Myc-TB analysis on 109 sputum samples from Djibouti and other analysis tools fed with WGS data from corresponding cultures are shown. ^#^: two rifampicin resistance phenotypes predicted by MTBSeq and PhyResSE and/or Mykrobe based on *rpoB* S431T and D435V, reflecting probable WGS or culture contaminations (see text); ^¶^: seven pyrazinamide resistance phenotypes predicted for “*Mycobacterium canettii*”-containing cultures by MTBSeq and Mykrobe based on *panD* M117T and *pncA* A46A, respectively; ^+^: one pyrazinamide resistance phenotype predicted by MTBSeq based on *pncA* D136G; ^§^: 11 resistant phenotypes predicted by Deeplex Myc-TB based on 10 minority variants (3–12%) and one *ethA* frameshift-causing indel; ^ƒ^: two streptomycin resistance phenotypes predicted by Deeplex Myc-TB and Mykrobe based on *gidB* G69D; ^##^: two ethambutol resistance phenotypes predicted by Deeplex Myc-TB and PhyResSE based on *embB* S297A and Y319S.

With the 1150 predicted phenotypes by Deeplex Myc-TB (155 resistant and 995 susceptible), the mean sensitivity and specificity *versus* available MTBSeq predictions was 93.5% and 98.5%, respectively (table 4). 108 additional phenotypes (8.6%) were not predicted by Deeplex Myc-TB due to the presence of uncharacterised mutations. Agreement on resistance prediction was 100% for all applicable drugs, except for rifampicin (93.5%) and pyrazinamide (71.4%). The rifampicin score resulted from probable differences between primary samples directly tested by Deeplex Myc-TB and cultures used for WGS analysis. Indeed, two resistance predictions by MTBSeq (and also by PhyResSE and Mykrobe) (figure 3) reflected detection of two minor resistance-associated variants in *rpoB* that were undetected by Deeplex Myc-TB despite high coverage depths at these positions, suggesting genotypic heterogeneity/contamination that was introduced/amplified after sputum processing, during subsequent culturing or WGS (note S7 in the supplementary material). For pyrazinamide, the lower sensitivity of Deeplex Myc-TB *versus* MTBSeq mostly resulted from different interpretation of some sequence variants or different regions interrogated in “*M. canettii*” isolates, which are naturally resistant to pyrazinamide and locally prevalent in and highly restricted to Djibouti [35]. Deeplex Myc-TB did identify seven *M. canettii*-containing samples based on the phylogenetic SNP *pncA* A46A, but did not (then) explicitly predict resistance to pyrazinamide, in contrast to MTBSeq based on *panD* M117T (and Mykrobe, based on *pncA* A46A) (figure 3 and note S8 in supplementary material).

**TABLE 4:** Phenotype predictions by direct Deeplex Myc-TB analysis of DNA from 109 clinical specimens *versus* phenotype predictions by MTBSeq with whole-genome sequencing (WGS) data from culture

	**MTBSeq resistant**					**MTBSeq susceptible**					**All**		**Excluding uncharacterised**		**Uncharacterised** **%**
	**Deeplex Myc-TB n**				**Total n**	**Deeplex Myc-TB n**				**Total n**	**Sensitivity** **%**	**Specificity** **%**	**Sensitivity** **%**	**Specificity** **%**	
	**R**	**R_h_**	**S**	**U**		**R**	**R_h_**	**S**	**U**						
**Rifampicin**	29	0	2	1	32	0	0	75	2	77	90.6 (75.8–96.8)	100 (95.2–100)	93.5 (79.3–98.2)	100 (95.1–100)	2.8
**Isoniazid**	31	0	0	0	31	0	2	71	5	78	100 (89.0–100)	97.4 (91.1–99.3)	100 (89.0–100)	97.3 (90.5–99.2)	4.6
**Pyrazinamide**	18	2	8	3	31	0	3	73	1	77	64.5 (46.9–78.9)	96.1 (89.2–98.7)	71.4 (52.9–84.7)	96.1 (89.0–98.6)	3.7
**Ethambutol**	21	1	0	1	23	2	2	79	3	86	95.7 (79.0–99.2)	95.3 (88.6–98.2)	100 (85.1–100)	95.2 (88.3–98.1)	3.7
**Streptomycin**	14	0	0	0	14	2	2	35	23	62	100 (78.5–100)	93.5 (84.6–97.5)	100 (78.5–100)	89.7 (76.4–95.9)	30.3
**Fluoroquinolones**	0	0	0	0	0	0	1	104	4	109	NA	99.1 (95.0–99.8)	NA	99 (94.8–99.8)	3.7
**Amikacin**	5	0	0	0	5	0	0	67	1	68	100 (56.6–100)	100 (94.6–100)	100 (56.6–100)	100 (94.6–100)	1.4
**Kanamycin**	5	0	0	0	5	0	0	66	2	68	100 (56.6–100)	100 (94.6–100)	100 (56.6–100)	100 (94.5–100)	2.7
**Capreomycin**	12	0	0	0	12	0	0	59	4	63	100 (75.8–100)	100 (94.2–100)	100 (75.8–100)	100 (93.9–100)	5.3
**Ethionamide**	7	0	0	4	11	1	0	66	31	98	63.6 (35.4–84.8)	99.0 (94.4–98.2)	100 (64.6–100)	98.5 (92.0–99.7)	32.1
**Linezolid**	0	0	0	0	0	0	0	87	3	90	NA	100 (95.9–100)	NA	100 (95.8–100)	3.3
**Bedaquiline**	0	0	0	0	0	0	0	99	10	109	NA	100 (96.6–100)	NA	100 (96.3–100)	9.2
**Clofazimine**	0	0	0	0	0	0	0	99	10	109	NA	100 (96.6–100)	NA	100 (96.3–100)	9.2
**Total**	142	3	10	9	164	5	10	980	99	1094	88.4 (82.6–92.5)	98.6 (97.7–99.2)	93.5 (88.5–96.5)	98.5 (97.5–99.1)	8.7

Data for sensitivity and specificity are presented as mean (95% CI); total sensitivity and specificity data are weighted means. Deeplex Myc-TB sequencing and WGS data originate from a report of a national tuberculosis drug resistance survey conducted in Djibouti [13]. Deeplex Myc-TB predictions were compared with WGS-based MTBSeq predictions separately for each drug, across isolates with this data available. R: detection of at least one fixed resistance-associated mutation; R_h_: detection of resistance due to a minority variant (heteroresistance); S: detection of mutations known not to be associated with resistance (phylogenetic, benign, synonymous) or no mutation detected; U: detection of at least one nonsynonymous uncharacterised mutation in the absence of a resistance-associated mutation; NA: not applicable.

Conversely, 15 out of the 995 (1.5%) phenotypes predicted as susceptible by MTBSeq (excluding uncharacterised phenotypes by Deeplex Myc-TB) were identified as resistant by Deeplex Myc-TB (table 4). Of these, 10 (66.6%) discordances were due to minority resistance-associated variants at 3.3–12.8% only detected in sputa by Deeplex Myc-TB (figure 3 and note S7 in the supplementary material). Importantly, eight out of these 10 minority variants co-occurred with one or more minority resistance and/or phylogenetic variants within an individual sputum. This further supports true-positive variant calls reflecting genuine mixed strains and/or combined heteroresistance detected by targeted deep sequencing, missed by WGS analysis due to lower coverage depth or potential culture bias. The five remaining discordant resistance predictions resulted from divergent variant interpretation, involving one *ethA* frameshift-causing indel (mechanistically expected to cause ethionamide resistance, see earlier), two debated *embB* mutations and a *gidB* mutation (n=2) associated with low-level streptomycin resistance [18] (note S7 in the supplementary material), which were associated with resistance by both Deeplex Myc-TB and PhyResSE and/or Mykrobe, but not by MTBSeq (figure 3).

The mean sensitivity and specificity of the phenotypes predicted by Deeplex Myc-TB *versus* available PhyResSE/Mykrobe predictions was 98.5/93.1% and 97.2/95.3%, respectively (supplementary tables S12 and S13, and note S8 in the supplementary material,).

Although patient sputa were (presumably) all smear-positive as per study design, the rate of successful Deeplex Myc-TB sequence analysis *versus* microscopic grade was not investigated in the Djibouti survey. This parameter was therefore evaluated on a set of 1494 direct sputum samples from a nationwide survey conducted in the DRC [22]. Yield of culture from cetylpyridinium chloride-stored sputum largely suffered from transport delays, whereas ethanol-preserved sputum samples were kept at room temperature for subsequent DNA extraction using a modified Maxwell 16 Low Elution Volume DNA Purification System [22]. Therefore, culture-free Deeplex Myc-TB testing was used mostly as a stand-alone assay for extensive pDST in TB patients, after their inclusion based on Ziehl–Neelsen smear positivity and positive *M. tuberculosis* detection on Xpert MTB/RIF.

**FIGURE 4:**
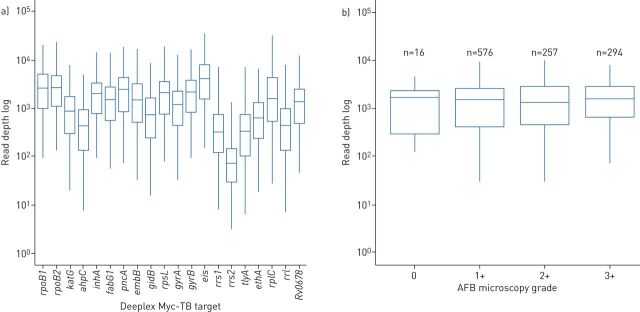
Log read depth obtained by direct Deeplex Myc-TB testing of DNA extracted from clinical specimens collected in a tuberculosis drug resistance survey conducted in the Democratic Republic of the Congo: a) log read depth at each drug resistance-associated Deeplex Myc-TB target on the total set of 1494 sputum samples and b) log read depth at Deeplex Myc-TB targets according to acid-fast bacilli (AFB) microscopy grading of 1143 sputum samples with available microscopic examination data. Box and whisker plots show median with interquartile range (IQR) and minimum–maximum range with a maximum of 1.5 IQR, respectively .

Of the 1143 sputum samples with available microscopic examination data, mean pan-target read depth exceeded 1000× for samples graded 1+, 2+ and 3+, although, as expected, the dispersion towards lower values was inversely correlated with microscopic grading (figure 4a and b, and supplementary table S14). Broadly similar read depths were also observed for samples that were graded negative (n=16 only, as per the standard survey design normally enrolling smear-positive TB patients only) or without reported grading (n=351). Despite the indeterminate bacterial loads in a third of the 1494 samples, MTBC was identified in 1258 (84.2%) of them. Of the 19 422 expected phenotypes in the 1494 samples, 73.5% (14 277) were automatically predicted (80.7–82.4% for the four first-line drugs), based on detected resistance mutations or absence of both resistance and uncharacterised mutations with minimal 5× read depth over ≥95% of the reference targets. Uncharacterised mutations were detected for an additional 5.9% (n=1137) and 625 additional susceptible phenotypes (3.2%) could be predicted after verification of effective minimal coverage of all resistance positions in the corresponding target. When distinguished by microscopic grade, the proportion of predicted phenotypes in samples without microscopy results (81.1%) was actually higher than for samples that were graded 0 (67.8%), 1+ (69.4%), 2+ (69.5%) and 3+ (76.3%).

Phenotypes for aminoglycosides, and to a lower extent linezolid, were relatively less frequently predicted, as a reflection of comparatively lower average read depths on *rrs* and *rrl* rDNA targets (supplementary table S14), expectedly due to competing commensal rDNA reads. However, such competition does not detectably affect specific calling of variants in samples with well-covered targets, as seen from the high degree of concordance with variants detected by WGS analysis on culture in the Djibouti dataset (except for minority variants only detected by deep sequencing that are likely true in most cases; note S6 in the supplementary material), and from full agreement between predictions of susceptible phenotypes for aminoglycosides and linezolid with those made by WGS analysis in the same dataset (table 4).

## Discussion

Based on a large dataset, our results show a high degree of accuracy of the Deeplex Myc-TB assay for extensive prediction of both susceptibility and resistance to anti-tuberculous drugs with an efficiency close to WGS, directly achievable at least from AFB smear-positive clinical specimens. In contrast, susceptibility in particular cannot be reliably inferred from a negative result with conventional molecular tests, because of the limited set of resistance-conferring mutations covered [14].

Importantly, most of the residual predictions of Deeplex Myc-TB discordant with susceptible or resistant phenotypes in the tested set of 429 MTBC isolates involved pyrazinamide, ethambutol and ethionamide, for which pDST is an imperfect standard [3, 31]. Likewise, for isoniazid or rifampicin, the sole discrepant resistant predictions by Deeplex Myc-TB all involved low-level resistance variants, frequently missed by liquid pDST, although they are critical to capture to avoid unfavourable treatment outcome or relapse [3, 32, 33]. For these reasons, sequencing of relevant genes is now proposed as a reference, at least for rifampicin, pyrazinamide, ethambutol and ethionamide [31]. Deeplex Myc-TB can thus be considered as outperforming the pDST standard for these drugs.

Crucially in this respect, the agreement with WGS analysis was almost complete in both the *in silico* and experimental datasets. All heteroresistant calls detected by both methods in the set of 429 MTBC strains were concordant with resistance phenotypes. The sole heteroresistant variant exclusively detected (at 4.9%), in an ethambutol-susceptible phenotype, by deeper sequencing with Deeplex Myc-TB involved an e*mbB* M306V variant. Variants in this *embB* 306 codon (as well as codons 354, 406 and 497) are assumed to be resistance mutations, regardless of the pDST result obtained [36]. Likewise, most of the resistance predictions made only by direct Deeplex Myc-TB analysis on sputa from the Djibouti survey were due to resistance minority variants, undetected by MTBSeq/PhyResSE/Mykrobe in WGS data likely due to insufficient read depths and/or culture bias eliminating potentially less fit (resistant) subpopulations [37] before subjection to WGS.

The optimal read depth-dependent limit of 3% for detecting minority populations with this Deeplex Myc-TB version was defined to secure true variant calls, after systematic analysis of sequence noise levels across target positions. This limit is substantially better than those of conventional molecular tests [38], although not as sensitive as the 0.1–1% limit claimed for the TGen deep-sequencing assay [10, 39]. While we recently developed an enhanced detection mode capable of reliably detecting minority variants down to 1%, this can only be reached on hypercovered target portions. Going below this threshold results in an unacceptably higher rate of false-positive base calls, affecting the overall specificity of such assays (data not shown). A genetic cut-off around this value seems reasonable, as old studies using the phenotypic proportion method showed that therapeutic success was unlikely above 1% of growing drug-resistant bacilli [40]. However, the correspondence between phenotypic and genetic estimates could be influenced by the uncertainty of phenotypic proportions given the clumping nature of mycobacteria and/or fitness cost of some resistance mutations resulting in underrepresentation of the cultured resistant subpopulation [37]. More studies are needed to address this important question.

Despite its large dataset, this study has limitations. The reference collection of 429 strains included relatively few resistant phenotypes for the second-line drugs investigated (none for bedaquiline and linezolid). Nevertheless, as also partly suggested from our *in silico* analysis, the Deeplex Myc-TB targets and mutation catalogue comprise the main or even the exclusively established genomic targets and most determinants of resistance to these drugs in clinical isolates (*e.g. gyrA* and *gyrB* quinolone resistance-determining regions for the fluoroquinolones). pDST data were not available for clinical specimens from Djibouti and the DRC, as pDST is not routinely performed on all newly diagnosed TB patients in such resource-limited countries, and because of the difficulty to re-culture samples even with preservatives added upon storage and transportation. However, our results showed closely matching phenotype predictions between Deeplex Myc-TB and the ultimate genotypic reference, *i.e.* WGS analysis, on the Djibouti dataset. Finally, the number of tested smear-negative samples clearly identified as such in the DRC dataset was limited. Nevertheless, the established limit of detection of 100–1000 extracted genome copies indicates that the test can be applied on all smear-positive samples and at least part of smear-negative samples with any reasonably efficient DNA extraction method. Consistently, the similar mean read depths obtained on the specimens graded 1+, 2+ and 3+ from the DRC survey also suggest a limit of detection ≤1+ (corresponding to 5000–10 000 genomes·mL^−1^ of sample) with the DNA extraction conditions used, accounting also for the fact that the equivalent of about a tenth of the available 1–5 mL of the DRC samples was generally used for DNA extraction and testing. Further consistent with such analytical sensitivity, in a recent study using QIAamp DNA Mini kits for DNA extraction, complete Deeplex Myc-TB phenotypic predictions could be made from all 37 smear-positive (including five scanty) and two smear-negative samples, in a pilot series of 50 clinical specimens [41].

In conclusion, the results of this extensive evaluation demonstrate the potential of the Deeplex Myc-TB assay to reliably guide personalised TB treatment, from culture or directly from clinical specimens depending on their bacterial loads. As the test can also be used on Illumina iSeq100 and MiniSeq, in addition to the MiSeq and NextSeq platforms used here, the scalability of throughputs, with optimal batches of 16 (iSeq100) to 384 tests per run (NextSeq) (including three controls per run), can cover the needs of many clinical laboratories at local/regional or nationally centralised levels. Its use might be particularly cost-effective upon positive MTBC detection (with or without rifampicin resistance) with a more sensitive rapid triage test such as Xpert MTB/RIF. Particularly when used on sputum, this assay will significantly reduce the total turnaround for generating extended DST reports. Culturing could potentially be restricted to samples without conclusive Deeplex Myc-TB results and pDST potentially limited to drugs for which uncharacterised mutations (instead of resistance, benign or no mutation) are detected in the relevant genes, as proposed for WGS-based phenotype predictions from culture [3], or for the new/repurposed drugs bedaquiline, clofazimine and delamanid/pretomanid, for which resistance mutations are poorly characterised (*Rv0678* for bedaquiline/clofazimine) or not (yet) covered by the assay (*atpE* and *pepQ* for bedaquiline and all (candidate) genes for delamanid and pretomanid). Planned improvements include addition of such extra targets.

## Supplementary material

10.1183/13993003.02338-2020.Supp1 **Please note:** supplementary material is not edited by the Editorial Office, and is uploaded as it has been supplied by the author.
Supplementary notes ERJ-02338-2020.Supplement
Supplementary tables ERJ-02338-2020.Tables
Supplementary figure S1 ERJ-02338-2020.Figure_1
Supplementary figure S2 ERJ-02338-2020.Figure_2
Supplementary figure S3 ERJ-02338-2020.Figure_3
Supplementary figure S4 ERJ-02338-2020.Figure_4
Supplementary figure S5 ERJ-02338-2020.Figure_5
Supplementary figure S6 ERJ-02338-2020.Figure_6

## Shareable PDF

10.1183/13993003.02338-2020.Shareable1 This one-page PDF can be shared freely online.
Shareable PDF ERJ-02338-2020.Shareable
