# Supplementary material for: Deep amplicon sequencing for culture-free prediction of susceptibility or resistance to 13 anti-tuberculous drugs
Source: Eur Respir J. 2021 Mar 18;57(3):2002338. doi: 10.1183/13993003.02338-2020 (PMC8174722; doi:10.1183/13993003.02338-2020)
Supplement: Supplementary file 1 [file ERJ-02338-2020.Supplement.pdf]

Supplementary material	
Paper title	Deep amplicon sequencing for culture-free prediction of susceptibility or resistance to 13 anti-tuberculous drugs
Authors	Agathe Jouet, Cyril Gaudin, Nelly Badalato, Caroline Allix-Béguec, Stéphanie Duthoy, Alice Ferré, Maren Diels, Yannick Laurent, Sandy Contreras, Silke Feuerriegel, Stefan Niemann, Emmanuel André, Michel K Kaswa, Elisa Tagliani, Andrea Cabibbe, Vanessa Mathys, Daniela Cirillo, Bouke C de Jong, Leen Rigouts, Philip Supply
PMID	

S1. Limit of detection for MTBC or NTM identification

As Deeplex Myc-TB does not depend on a specific DNA extraction method, the assay's limit of detection for MTBC identification was estimated in terms of number of extracted MTBC genome copies by performing serial dilutions of purified, pre-quantified genomic DNA from three well-characterized MTBC strains, and a mixture of two strains at a 5-95% ratio. Four independent amplification replicates (each with a different kit lot) and sequencing rounds were performed for each dilution, yielding a total of 16 tests per dilution level (10, 10^2, 10^3 and 10^4 genomes). Based on *hsp65* sequence best match analysis, all samples were identified as *M. tuberculosis* at 10^4 and 10^3 genomes, 13/16 samples (81.2%) at 10^2 genomes while none were identified with 10 genomes (Supplementary Figure S1).

Likewise, all three independent replicated analyses of 10^2, 10^3 and 10^4 genomes of *M. intracellulare* resulted in the expected 100% identification based on *hsp65* best match analysis, while two of three replicates with 10 genomes generated this expected result (Supplementary Figure S2).

S2. Assay design

The assay is based on a single 24-plex PCR targeting 18 major genes associated with resistance to 13 first- and second-line anti-TB drugs/drug classes, amplified in 21 amplicons (Figure 1 and Table 1 in the main text). Two separate amplicons cover

rpoB codons 384-516 and 158-335, including not only the rifampicin resistance determining region but also other regions containing known rifampicin resistance mutations that are missed by rapid molecular tests (*e.g.* I491F [1,2] and V170F [3]). For genes such as *pncA* and *Rv0678*, associated with pyrazinamide and bedaquiline/clofazimine resistance respectively, where resistance mutations are known to arise across the entire corresponding sequences, complete coding sequences along with part of promoter regions are covered by a single amplicon. For *ethA*, associated with ethionamide resistance, two overlapping amplicons cover the gene and part of the promoter. A 401-bp segment of the *hsp65* gene is used as primary reference for mycobacterial species identification [4], the direct repeat region for spoligotype identification of MTBC strains [5], and an internal control sequence to control PCR inhibition.

After PCR and amplicon library preparation and sequencing, reads from FASTQ files uploaded to the Deeplex Myc-TB web application are automatically mapped on *M. tuberculosis* H37Rv reference sequences using Bowtie 2 [6]. Variants are called with a limit of 3% read proportion depending on coverage depth at the position (134x required at least), using proprietary scripts and filters. Detected variants are automatically associated with drug resistance or susceptibility, or phylogenetic SNPs (*i.e.* MTBC (sub)lineage-defining) by comparison with integrated reference variant databases (Table 2 in the main text). Priority is given to the collaborative, curated database ReSeqTB when detected variants are included in this database [7]. When not included in these databases, mutations are defined as uncharacterized. Such categorization for off-catalogue mutations, instead of explicit or implicit prediction of susceptibility as currently done by other tools [8–10], has been shown to be an effective flag to avoid some very major errors (missed resistance) and trigger

subsequent pDST for confirmation [11]. Mycobacterial species and spoligotype are identified using additional integrated databases, based on best-match against *hsp65* sequences from 167 mycobacterial species/subspecies derived from data from Dai et al. [4] and *M. kansasii* and *M. chelonae*-specific mutations in *rrs* and *rrl*, and “SIT” types from the global SITVIT database [12], respectively. The publication from Dai et al. was selected as a primary reference for NTM identification because the majority of the *hsp65* sequence entries were distinctively curated by validation with type strains or were supported by multiple GenBank records deposited by independent research groups.

For some datasets (i.e. obtained from isolates from Sciensano and the TDR collection, clinical specimens from Djibouti, and a subset of clinical specimens from the Democratic republic of Congo), a beta version of the Deeplex Myc-TB kit was used, which differed from the up-to-date version (including the targets described in Table 1 in the main text) by not yet including the clofazimine/bedaquiline resistance-associated target *Rv0678* and the *ahpC* promoter region including two rare isoniazid resistance-associated mutations, namely G-48A and C-57T. To verify that the coverage depths on the other targets did not differ between the two versions, the mean read depths on these targets on 6 replicates of the same *M. bovis* BCG positive control obtained either with the first or the second versions were compared. After verifying the normality of the data with Q-Q plots using R, no significant difference was found ($p>0.05$) by using a non-parametric test (Wilcoxon) between the log mean read depths obtained with the first and the second versions (see the figure below).

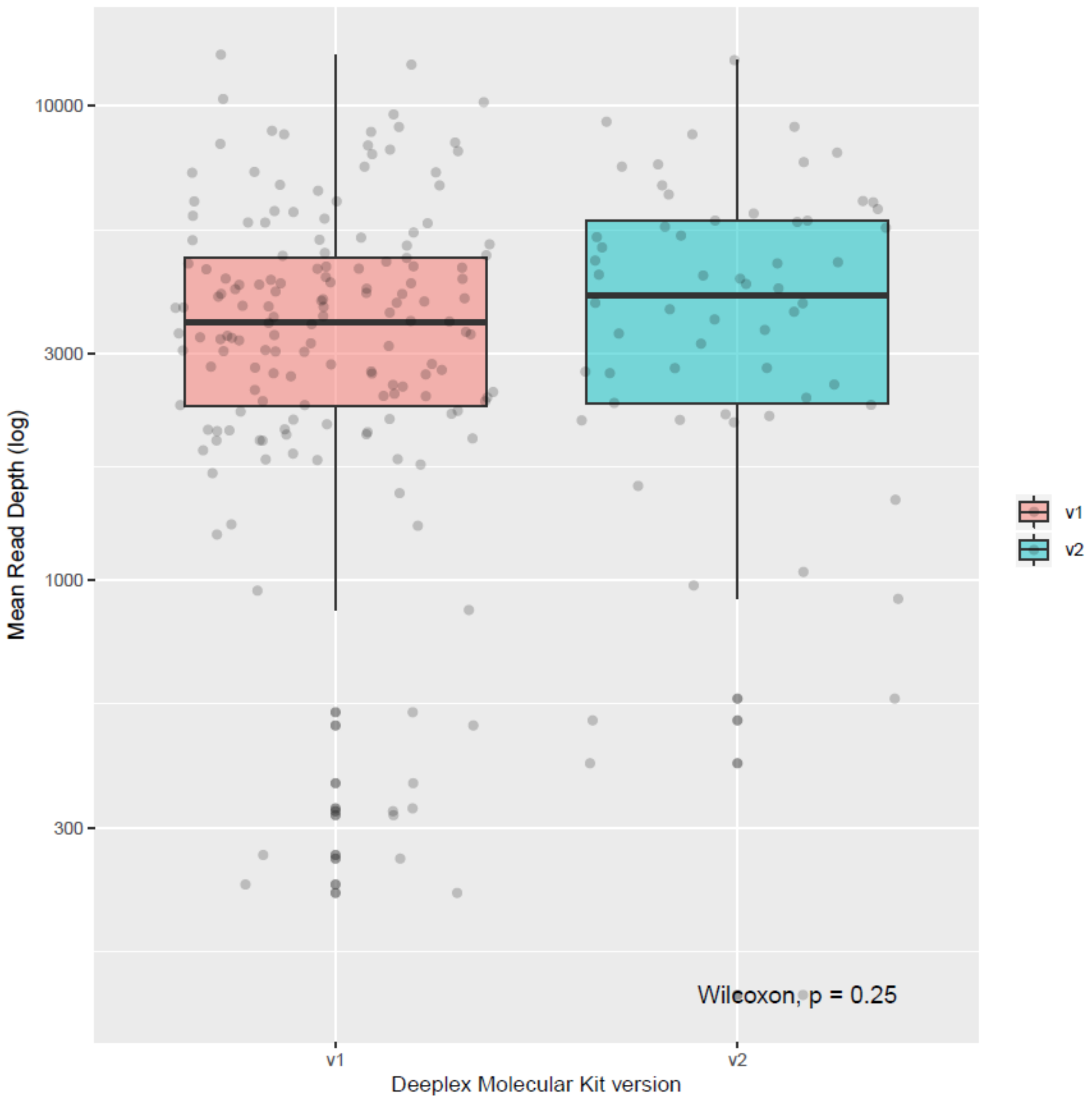

S3. Mycobacterial species identification

For extensive testing of mycobacterial species identification, DNA extracts from 370 nontuberculous mycobacterial (NTM) strains from the Institute of Tropical Medicine of Antwerp (ITM, Belgium) were used, including 3 strains used in routine testing at the ITM, 30 type strains and 337 strains from the Belgian Culture Collection of Microorganisms (BCCM), maintained under quality control at ITM. These represented 73 different NTM species/species complexes, identified at the ITM based on a combination of phenotypic testing, *rpoB* and/or *16S* rDNA Sanger sequencing. Multiple strains from the major clinically relevant taxa were purposely included to

represent a potential intraspecies diversity. After application of the assay on these DNA extracts, the Deeplex Myc-TB web application was used for automated best-match analysis using *hsp65* as a primary gene target, complemented by *rrs* and *rrl* sequence analysis for some mycobacterial species (Supplementary Table S2).

Out of these 370 strains, 292 strains were identified at (sub)species or species complex levels by both reference and Deeplex Myc-TB testing (Supplementary Figure S3, Supplementary Table S2). Of these, 194 (66.4%) were best-matched at the same taxonomic level by both methods, while 76 (26%) were matched at the same complex level, with a sublevel identification by Deeplex Myc-TB (n=20; essentially *M. intracellulare* complex strains), or the reference (n=40; essentially *M. avium* complex strains, relatively overrepresented in this dataset, which are not further identified as (*avium* or *hominisuis*) subspecies by Deeplex Myc-TB), or showing a different subspecies (n=16) across methods. Among the 194 cases concordant at the same taxonomic level, two distinct *hsp65* sequence variants both best matching a same species were detected by Deeplex Myc-TB in 13 strains, indicating underlying clonal complexity (*e.g.* in 3 *M. nonchromogenicum* and 2 *M. smegmatis* strains). In addition, 4 of 292 (1.4%) strains had a partial match either because they were best matched between two possible equidistant species (n=1), or detected in a mixture with another species (n=3). Only 18 (6.2%) strains had taxonomically discordant results between both methods, *i.e.* without full or partial match as above. These mostly consist of single discordant cases among otherwise partially/fully concordant strains for a species (*e.g. M. ulcerans*, n=1/13; *M. kansasii*, n=1/14), or species rarely involved in infections (*e.g*. *M. peregrinum*; n=2) [13,14].

To note, for residual isolates with discrepant subspecies within a matching complex (e.g. *M. intracellulare vs M. chimaera)*, the correctness of the Deeplex Myc-TB

identification was actually often possible or probable. Indeed, in these cases, conflicting or ambiguous identifications were frequently seen between the *rpoB* and 16S rDNA reference probes, with one or the other partially or fully matching the Deeplex Myc-TB result (Supplementary Table S2). This held also true for some of few individual isolates (of otherwise well identified species) that were discordant even at complex level (e.g. *M. kansasii* vs *M. gastri*).

Of the remaining 78 strains out of the 370 analyzed in total, 5 had no identification by either method while the others were identified to (sub)species/complex level by Deeplex Myc-TB (n=28) or the reference (n=45) only. The latter mostly consisted of strains from species of rare clinical prevalence (including 5 other *M. peregrinum* or *M. septicum* strains), or reflect a minority of cases among strains otherwise partially/fully concordant for a species or complex (*e.g.* 6/16 *M. chelonae* complex, including 1/11 M. *chelonae subsp chelonae*). Only a few NTM species of relatively higher clinical significance had a majority of strains without Deeplex best matched species, including in particular some strains from the *M. terrae* complex: *M. arupense* (2/3 without best match), and *M. virginiense* (1/2; the other strain being best matched to *M. aurum*).

Altogether, these results thus indicate highly reliable identification of mycobacterial species by the Deeplex Myc-TB. The range of 167 mycobacterial (sub)species covered by the identification database, including virtually all clinically relevant ones, is substantively larger than that of conventional molecular tests, such as line probe assays covering 46 (sub)species in two separate assays [15], and is close to that of MALDI TOF mass spectrometry-based methods, such as Bruker's (Billerica, Massachusetts, USA) reportedly covering 178 (sub)species (https://www.bruker.com/products/mass-spectrometry-and-separations/maldi-

biotyper-for-microbiological-research/mycobacteria/overview-mycobacteria.html). We plan to further increment our identification database by first basing on the comprehensive, updated list of taxa with a validly published and correct name, included in the Prokaryotic names with Standing in Nomenclature (LSPN) database (https://www.bacterio.net/genus/mycobacterium). While the number of such taxa (excluding synonyms) as of August 2020 is 192 in this list, most of those not (yet) included in our database concern relatively novel species described by a single research group thus far, and based on very few or even just one strain(s) in most cases. As was done in the work from Dai et al. [4] (see above), we will prioritize further incorporation of new taxa that are described by independent research groups.

Of final note, the detection of distinct subpopulations of variant *hsp65* reads within some samples of the NTM strain collection (Supplementary Table S2) as well as in some clinical specimens from Djibouti (see below and Supplementary Figure S4), indicate distinct co-existing species (NTM or NTM with MTBC) or species variants. Detection of co-infecting or co-colonizing mycobacterial species is important for correct interpretation of available pDST results and guidance of patient treatment [16].

S4. Deeplex Myc-TB phenotype prediction versus phenotypic testing

We compared the ability of both Deeplex Myc-TB and Illumina-based WGS analysis to detect variants, using a total of 429 strains. For this comparison, we used a state-of-the-art WGS analysis pipeline, validated for accurate SNP calling by using short read data sets derived from isolates with known complete genomes in accordance with recent guidelines [17,18]. Mean on-target coverage depth by Deeplex Myc-TB was 3,171x (SD=3691.7) (Supplementary Figure S5, Supplementary Table S15), with a minimum mean of 600x on *rrs2*, which largely exceeds the minimal read depth set

to reach the maximal limit of detection of 3% for minority variants (*i.e*. >=134x). Mean reference coverage breadth was 99% (SD=4.9). In comparison, mean coverage depth and breadth on the same targets by WGS was 71x (SD=70) and 98.9% (SD=6.2), respectively.

In total, 2403 variants were identified in the Deeplex Myc-TB targets (2323 SNPs, 65 deletions and 15 insertions), including 798 resistance-associated variants. Of all variants, 2349 (97.8%; including 2269 SNPs and all 80 indels), including 779 (97.6%) of the resistance variants, were detected by both Deeplex and our WGS analysis pipeline initially used with a common high-frequency threshold (85%) used for SNP calling. The remaining 54 (2.2%) SNPs were all variants with frequencies below this threshold (Supplementary Table S4). Of these, 34 (63.0%) were found as co-occurring with 1 to 7 other such variants within an individual isolate (involving resistance and/or phylogenetic variants in 26 cases), indicating mixed strains and/or combined heteroresistance and thus genuine genotypic subpopulations detected by targeted deep sequencing. Moreover, about half of the total 54 (n=24) were identified in WGS-derived reads when analyzed under low frequency detection mode, detected with similar ~5-80% frequencies as in Deeplex Myc-TB-derived reads in virtually all cases. The other half (n=30), undetected even under these conditions, concentrated those with lowest frequencies of ~3-10% only identified by targeted deep sequencing, as an expected reflection of the lower coverage depth obtained with WGS (Supplementary Table S4). Of these, only one (at a frequency of 4.9%) corresponded to a resistance-associated mutation (to ethambutol; see main text).

Of the 1583 susceptible phenotypes with prediction, only 41 (2.6%) were discordantly predicted as resistant. Among these, 28 (68.3%) involved ethambutol and *embB* mutations known to be associated with poor phenotypic reproducibility (*e.g.* 12

M306V, 7 M306I and 6 G406D [19], including two minority variants, one of which was not detected by WGS (4.9%)). Six discordances consisted of indels (all confirmed by WGS) causing frameshifts in *ethA*, all mechanistically expected to cause ethionamide resistance due to defective drug activation, as a result of impaired encoding of the monooxygenase EthA [20]. Also meaningful in this respect, four other *ethA* frameshifts were found in ethionamide-resistant phenotypes, in line with notorious phenotypic variability for this drug [21]. The only three discordances for isoniazid involved *inhA* S94A (without *fabG1* C-15T) in two isolates and *ahpC* G-48A in one isolate, showing variable association with (low level) isoniazid resistance among different studies [7,19,22]. Likewise, the only three discordant predictions for rifampicin involved *rpoB* L452P, H445N and D435Y, all part of the so-called “disputed” mutations with low rifampicin resistance levels frequently missed by liquid pDST, yet associated with poor clinical outcome on rifampicin-based treatment [23,24]. Lastly, the *gidB* A138V mutation, found in one streptomycin-susceptible isolate and associated with streptomycin resistance by Deeplex Myc-TB, is also subject to conflicting association with streptomycin resistance [7,25].

S5. Interpretation of uncharacterized mutations conditional on isoniazid susceptibility results

Because susceptibility to isoniazid predicts susceptibility to other first-line drugs [26] and as described for WGS-based prediction [27], the overall predictability of the test was further improved when a predicted susceptibility to isoniazid was considered to predict susceptibility to other first-line drugs, even when uncharacterized mutations were present in gene targets relevant for these drugs. This reduced the proportions of uncharacterized predictions from 1.9-4.4% to 0.5-2.5% for rifampicin, ethambutol and pyrazinamide, with correct re-classification as susceptible phenotypes in 5, 3 and

1 cases, respectively (Supplementary Table S7). To note however, this also resulted in the incorrect prediction of one additional susceptible phenotype (i.e. for a resistant phenotype) for each of these drugs.

S6. Variant detection by Deeplex Myc-TB on clinical specimens versus WGS analysis

We compared variant detection and phenotype predictions based on available Deeplex Myc-TB sequencing data directly obtained from 109 clinical specimens from a nationwide survey conducted in Djibouti, versus analysis of WGS data obtained after culturing [28]. DNA was extracted from these sputum samples as per a protocol described in the Deeplex-MycTB user's manual, as follows. After decontamination by using N-acetyl-L-cysteine combined with sodium hydroxide and sodium citrate procedure, utilizing NACPAC™RED kit from AlphaTec (Vancouver, Washington state, USA), followed by treatment by NPC67 neutralizing buffer and resuspension in 0.5 ml – 1 ml of PRB pellet resuspension buffer, both from AlphaTec, 100 µl to 500 µl of the suspensions were transferred in a screw cap or safe-lock microfuge tube and heat inactivated at 95°C for 30 min in an oven. After subsequent centrifugation at 20,000 g for 30 min, the supernatant was removed and discarded, and 250 µl of 10 mM Tris-HCl pH 7.8 was added, followed by brief vortexing, incubation at 95°C for 15 min and brief spinning down to collect potential droplets in tube cap. The entire volume (avoiding the pellet) was transferred in a new tube containing molecular biology grade 0.15 mm zirconium beads (Next Advance, Troy, New York, USA) in 2ml microtubes, vortexed at least for 30s, using a benchtop vortex mixer at full speed, followed by brief spinning down and incubation at least for 30 min at -20°C. After thawing at room temperature, the supernatant was transferred in a new 1,5 ml microtube, by avoiding resuspension of beads. A DNA concentration step was then

performed by adding 1 µl of 20 mg/ml glycogen solution (Sigma Aldrich, Saint-Louis, Missouri, USA) and mixing, 0.1 volume of 3 M sodium acetate at pH 5.2 and mixing, and 3 volume 100% pre-cooled ethanol, followed by vigorous vortexing for 10s, incubation at -20°C for 10 min, and centrifugation at 15,000 g for 20 min. After discarding of the supernatant, 600 µl of freshly prepared, pre-cooled 70% ethanol were added, followed by centrifugation at 15,000 g for 5 min. After discarding the supernatant, the pellet was air dried for 15 min and resuspended in 20 µl of sterile water.

Overall, 693 of 752 total variants (92.2%) were detected by both Deeplex Myc-TB and our WGS pipeline used under low frequency detection mode. Apart from one fixed *pncA* SNP not detected by Deeplex Myc-TB, 59 SNPs were completely missing from WGS reads even when analyzed under low frequency detection mode. All latter SNPs were minority variants with frequencies from 3 to 25% in most cases, clustering in 20 samples. Of these 59, 45 were, or co-occurred at similar low frequencies with, resistance and/or phylogenetic variants within a sample, suggesting likely true variant calls missed by a generally (much) lower read depth by WGS (Supplementary Table S11). Of the remaining 14 SNPs, some could possibly reflect Deeplex Myc-TB amplification of false positive SNPs occasionally seen in test samples with low genome copy numbers (Supplementary Figure S6). However, when occurring, such sporadic effect typically affects only one to few uncharacterized or synonymous minority SNPs – representing the vast majority of possible nucleotide changes across all targets- at frequencies close to the 3% limit of detection (Supplementary Figure S6).

Of note, within some of these clinical specimens from Djibouti, Deeplex Myc-TB detected distinct subpopulations of variant *hsp65* reads (Supplementary Figure S4),

indicating distinct co-existing species (NTM with MTBC). Detection of such NTM/MTBC co-infections has diagnostic and clinical implications [29].

Moreover, potential/probable mixed infections with *M. tuberculosis* and "*M. canettii"* or strains of distinct MTBC lineages strains were detected by Deeplex MycTB in 8/109 (7.3%) sputum samples from Djibouti (and 37/1494 (2.3%) sputum samples from DRC), based on mixed phylogenetic SNP calls, possibly supplemented by the observation of a biphasic distribution of read depths on spoligotype spacers. This detection of mixed infections is important for epidemiological inferences, and may be as well of clinical relevance, as mixed infections have been associated with poor tuberculosis treatment outcomes, also independently of heteroresistance [30].

S7. Deeplex Myc-TB on clinical specimens vs MTBSeq-based predictions

The sensitivity of 93.5% for rifampicin of Deeplex Myc-TB versus MTBSeq on the Djibouti dataset resulted from two resistance predictions by MTBSeq (also by PhyResSE and Mykrobe; Figure 3 in main text) based on the detection of two minor variants in *rpoB* (S431T at 42.7%, and D435V at 25.9%) that co-occurred with one or more minor variants in other genes (Deeplex Myc-TB targets as well as other genes) sequenced by WGS. None of these minor variants were detected in the two corresponding sputa analyzed by Deeplex Myc-TB (even before filtering of the sequence data) despite high coverage depths at these positions, suggesting genotypic heterogeneity or contamination, introduced or amplified during subsequent culturing or WGS processing.

Fifteen of the 995 (1.5%) phenotypes predicted as susceptible by MTBSeq in the Djibouti dataset (excluding uncharacterized phenotypes by Deeplex Myc-TB) were identified as resistant by Deeplex Myc-TB (Table 4 in main text). Of these, 10 (66.6%) discordances were due to minority resistance-associated variants at 3.3-

12.8% detected in sputa by Deeplex Myc-TB, but missed by MTBSeq, PhyResSE and Mykrobe (Figure 3; *katG* S315T (n=2); *embB* G406A and co-occurring G406A + P404S; *pncA* D8G, H57D, H71Y; *gyrA* D94A, and *rrs* A514C (n=2)). The five remaining discordant resistance predictions resulted from divergent variant interpretation, involving one *ethA* frameshift-causing indel (mechanistically expected to cause ethionamide resistance, see above), two debated *embB* mutations (S297A, Y319S [35]) and the *gidB* G69D mutation (n=2, associated with low-level streptomycin resistance [25]), which were associated with resistance by both Deeplex Myc-TB and PhyResSE and/or Mykrobe, but not by MTBSeq

S8. Deeplex Myc-TB on clinical specimens vs PhyResSE and Mykrobe WGS-based predictions

With the 950 predicted phenotypes by Deeplex Myc-TB (137 resistant, 813 susceptible), the mean sensitivity and specificity versus available PhyResSE predictions were 98.5% and 97.2% (Supplementary Table S12). Ninety additional phenotypes (8.7%) were not predicted by Deeplex Myc-TB due to the presence of phenotypically uncharacterized mutations. Agreement on resistance prediction was 100% for all applicable drugs, except for rifampicin (93.5%). The latter score resulted from two PhyResSE resistance predictions based on the detection of two minor variants in *rpoB* (S431T at 33%, and D435V at 9%) that co-occurred with one or more minor variants in other genes (Deeplex Myc-TB targets as well as other genes) sequenced by WGS. None of these minor variants were detected in the two corresponding sputa analyzed by Deeplex Myc-TB (before or after filtering of the data) despite high coverage depths at these positions, suggesting genotypic heterogeneity, or contamination introduced or amplified during subsequent culturing or WGS processing.

Conversely, 23 of the 813 (2.8%) phenotypes predicted as susceptible by PhyResSE were identified as resistant by Deeplex Myc-TB (Supplementary Table S12). Of these, 11 (47.8%) were due to fixed variants or indels detected but not regarded as associated with resistance by PhyResSE. Four mutations (2 T160A, 2 K96N) and 6 frameshift-causing indels or large deletions in *pncA* were found in samples predicted to be MDR with additional resistance to at least ethambutol, in five out of six cases, thus supporting probable pan-first-line resistance (including pyrazinamide). Likewise, one *ethA* frameshift-causing indel was mechanistically [20] predicted to cause resistance by Deeplex Myc-TB and not by PhyResSE. Finally, 12 (52.2%) discordances were due to minority resistance-associated variants at 3.3-12.8%, detected by Deeplex Myc-TB but missed by WGS due to lower read depths (*katG* S315T (n=2); *embB* G406A and co-occurring minor G406A + P404S; *pncA* D8G, H57D, H71Y (n=2) and 1 frameshift-causing insertion; *gyrA* D94A, and *rrs* A514C (n=2)).

The overall proportion of predicted resistance by Deeplex Myc-TB relatively to Mykrobe was somewhat negatively skewed by the individual sensitivity of 58.8% for pyrazinamide, in contrast to 96.6%-100% for all other applicable drugs (Supplementary Table S13). This lower score resulted from the local prevalence of "*M. canettii"* isolates in Djibouti, which are naturally resistant to pyrazinamide and highly restricted to the Horn of Africa [31–34]. While both analysis tools identified "*M. canettii"* in 7 samples based on a fixed synonymous phylogenetic SNP *pncA* A46A, Deeplex Myc-TB did not explicitly report a pyrazinamide resistance prediction on this basis, resulting in 10 pyrazinamide resistant phenotypes predicted by Deeplex Myc-TB versus 17 by Mykrobe. Other discordances resulted from a rare apparent non-sense *rpoB* D435X mutation in a sample and two rare *katG* indels, which were not

reported as rifampicin or isoniazid resistance associated by Deeplex Myc-TB at the time of the study.

Finally, 31 of the 705 (4.4%) susceptible phenotypes predicted by Mykrobe were identified as resistant by Deeplex Myc-TB (Supplementary Table S13). These include the 10 out of 12 phenotypes predicted based on resistance-associated minority variants with frequencies of 3.3-12.8%, and the 7 capreomycin resistant phenotypes due to the fixed *tlyA* N236K mutation. Likewise, 3 of the 5 indels or large deletions in *pncA* ignored by PhyResSE were also unaccounted-for pyrazinamide resistance by Mykrobe. The remaining discordant phenotypes were all due to (near-)fixed variants. These include 7 various mutations in *pncA* (of which 6 were found in samples predicted to be MDR with additional resistance to ethambutol at least, arguing for first-line pan-resistance), a D435A *rpoB* mutation with indeterminate rifampicin association with resistance according to ReSeqTB [7], as well as S297A and Y319S *embB* mutations [35,36] and a *rrs* A906G mutation that were also predicted as resistance-conferring by PhyResSE.

References

[1] Sanchez-Padilla E, Merker M, Beckert P, Jochims F, Dlamini T, Kahn P, et al. Detection of drug-resistant tuberculosis by Xpert MTB/RIF in Swaziland. N Engl J Med 2015;372:1181–2.

[2] Makhado NA, Matabane E, Faccin M, Pinçon C, Jouet A, Boutachkourt F, et al. Outbreak of multidrug-resistant tuberculosis in South Africa undetected by WHO-endorsed commercial tests: an observational study. Lancet Infect Dis 2018;3099.

[3] Heep M, Brandstätter B, Rieger U, Lehn N, Richter E, Rüsch-Gerdes S, et al. Frequency of *rpoB* mutations inside and outside the cluster I region in rifampin-resistant clinical *Mycobacterium tuberculosis* isolates. J Clin Microbiol 2001;39:107–10.

[4] Dai J, Chen Y, Lauzardo M. Web-accessible database of *hsp65* sequences from *Mycobacterium* reference strains. J Clin Microbiol 2011;49:2296–303.

[5] Kamerbeek J, Schouls L, Kolk A, Van Agterveld M, van Soolingen D, Kuijper S, et al. Simultaneous detection and strain differentiation of *Mycobacterium tuberculosis* for diagnosis and epidemiology. J Clin Microbiol 1997;35:907–14.

[6] Langmead B, Salzberg SL. Fast gapped-read alignment with Bowtie 2. Nat Methods 2012;9:357–9.

[7] Miotto P, Tessema B, Tagliani E, Chindelevitch L, Starks AM, Emerson C, et al. A standardised method for interpreting the association between mutations and phenotypic drug resistance in *Mycobacterium tuberculosis*. Eur Respir J 2017;50.

[8] Feuerriegel S, Schleusener V, Beckert P, Kohl TA, Miotto P, Cirillo DM, et al. PhyResSE: A web tool delineating *Mycobacterium tuberculosis* antibiotic resistance and lineage from whole-genome sequencing data. J Clin Microbiol 2015;53:1908–14.

[9] Bradley P, Gordon NC, Walker TM, Dunn L, Heys S, Huang B, et al. Rapid antibiotic-resistance predictions from genome sequence data for *Staphylococcus aureus* and *Mycobacterium tuberculosis*. Nat Commun 2015;6:10063.

[10] Kohl TA, Utpatel C, Schleusener V, De Filippo MR, Beckert P, Cirillo DM, et al.

MTBseq: A comprehensive pipeline for whole genome sequence analysis of *Mycobacterium tuberculosis* complex isolates. PeerJ 2018;2018:1–13.

[11] Hunt M, Bradley P, Lapierre SG, Heys S, Thomsit M, Hall MB, et al. Antibiotic resistance prediction for *Mycobacterium tuberculosis* from genome sequence data with mykrobe [version 1; peer review: 2 approved, 1 approved with reservations]. Wellcome Open Res 2019;4.

[12] Demay C, Liens B, Burguière T, Hill V, Couvin D, Millet J, et al. SITVITWEB - A publicly available international multimarker database for studying *Mycobacterium tuberculosis* genetic diversity and molecular epidemiology. Infect Genet Evol 2012;12:755–66.

[13] Asmar S, Rascovan N, Robert C, Drancourt M. Draft genome sequence of *Mycobacterium mucogenicum* strain CSUR P2099. Genome Announc 2015;3:6–7.

[14] Kamijo F, Uhara H, Kubo H, Nakanaga K, Hoshino Y, Ishii N, et al. A case of mycobacterial skin disease caused by *Mycobacterium peregrinum*, and a review of cutaneous infection. Case Rep Dermatol 2012;4:76–9.

[15] Mäkinen J, Marjamäki M, Marttila H, Soini H. Evaluation of a novel strip test, GenoType Mycobacterium CM/AS, for species identification of mycobacterial cultures. Clin Microbiol Infect 2006;12:481–3.

[16] DS Sarro Y, Kone B, Diarra B, Kumar A, Kodio O, B Fofana D, et al. Simultaneous diagnosis of tuberculous and non-tuberculous mycobacterial diseases: Time for a better patient management. Clin Microbiol Infect Dis 2018;3:1–8.

[17] Pouseele H, Supply P. Chapter 10 - Accurate Whole-Genome Sequencing-

Based Epidemiological Surveillance of *Mycobacterium tuberculosis*. In: Sails A, Tang Y-WBT-C and ET for the D of MI, Sails A, Tang Y-W, editors. Curr. Emerg. Technol. Diagnosis Microb. Infect., 2015, p. 359–94.

[18] Meehan CJ, Goig GA, Kohl TA, Verboven L, Dippenaar A, Ezewudo M, et al. Whole genome sequencing of *Mycobacterium tuberculosis*: current standards and open issues. Nat Rev Microbiol 2019;17:533–45.

[19] Walker TM, Kohl TA, Omar S V., Hedge J, Del Ojo Elias C, Bradley P, et al. Whole-genome sequencing for prediction of *Mycobacterium tuberculosis* drug susceptibility and resistance: A retrospective cohort study. Lancet Infect Dis 2015;15:1193–202.

[20] Baulard AR, Betts JC, Engohang-Ndong J, Quan S, McAdam RA, Brennan PJ, et al. Activation of the pro-drug ethionamide is regulated in mycobacteria. J Biol Chem 2000;275:28326–31.

[21] WHO. Technical Manual for Drug Susceptibility Testing of Medicines Used in the Treatment of Tuberculosis. 2018.

[22] Lempens P, Meehan CJ, Vandelannoote K, Fissette K, De Rijk P, Van Deun A, et al. Isoniazid resistance levels of *Mycobacterium tuberculosis* can largely be predicted by high-confidence resistance-conferring mutations. Sci Rep 2018;8:1–9.

[23] Miotto P, Cabibbe AM, Borroni E, Degano M, Cirilloa DM. Role of disputed mutations in the *rpoB* gene in interpretation of automated liquid MGIT culture results for rifampin susceptibility testing of *Mycobacterium tuberculosis*. J Clin Microbiol 2018;56:1–9.

[24] Torrea G, Ng KCS, Van Deun A, André E, Kaisergruber J, Ssengooba W, et al.

Variable ability of rapid tests to detect *Mycobacterium tuberculosis rpoB* mutations conferring phenotypically occult rifampicin resistance. Sci Rep 2019;9:1–9.

[25] Feuerriegel S, Oberhauser B, George AG, Dafae F, Richter E, Rüsch-Gerdes S, et al. Sequence analysis for detection of first-line drug resistance in *Mycobacterium tuberculosis* strains from a high-incidence setting. BMC Microbiol 2012;12.

[26] Manson AL, Cohen KA, Abeel T, Desjardins CA, Armstrong DT, Barry CE, et al. Genomic analysis of globally diverse *Mycobacterium tuberculosis* strains provides insights into the emergence and spread of multidrug resistance. Nat Genet 2017;49:395–402.

[27] Allix-Béguec C, Arandjelovic I, Bi L, Beckert P, Bonnet M, Bradley P, et al. Prediction of susceptibility to first-line tuberculosis drugs by DNA sequencing. N Engl J Med 2018;379:1403–15.

[28] Tagliani E, Hassan MO, Waberi Y, De Filippo MR, Falzon D, Dean A, et al. Culture and Next-generation sequencing-based drug susceptibility testing unveil high levels of drug-resistant-TB in Djibouti: Results from the first national survey. Sci Rep 2017;7:1–9.

[29] Hwang SM, Lim MS, Hong YJ, Kim TS, Park KU, Song J, et al. Simultaneous detection of *Mycobacterium tuberculosis* complex and nontuberculous mycobacteria in respiratory specimens. Tuberculosis 2013;93:642–6.

[30] Shin SS, Modongo C, Baik Y, Allender C, Lemmer D, Colman RE, et al. Mixed *Mycobacterium tuberculosis*-strain infections are associated with poor treatment outcomes among patients with newly diagnosed tuberculosis,

independent of pretreatment heteroresistance. J Infect Dis 2018.

[31] Fabre M, Koeck JL, Le Flèche P, Simon F, Hervé V, Vergnaud G, et al. High genetic diversity revealed by variable-number tandem repeat genotyping and analysis of *hsp65* gene polymorphism in a large collection of “*Mycobacterium canettii*” strains indicates that the *M. tuberculosis* complex is a recently emerged clone of "*M. canettii*". J Clin Microbiol 2004;42:3248–55.

[32] Gutierrez MC, Brisse S, Brosch R, Fabre M, Omaïs B, Marmiesse M, et al. Ancient origin and gene mosaicism of the progenitor of *Mycobacterium tuberculosis*. PLoS Pathog 2005;1:0055–61.

[33] Supply P, Marceau M, Mangenot S, Roche D, Rouanet C, Khanna V, et al. Genomic analysis of smooth tubercle bacilli provides insights into ancestry and pathoadaptation of *Mycobacterium tuberculosis*. Nat Genet 2013;45:172–9.

[34] Blouin Y, Cazajous G, Dehan C, Soler C, Vong R, Hassan MO, et al. Progenitor “*Mycobacterium canettii*” clone responsible for lymph node tuberculosis epidemic, Djibouti. Emerg Infect Dis 2014;20:21–8.

[35] Plinke C, Rüsch-Gerdes S, Niemann S. Significance of mutations in *embB* codon 306 for prediction of ethambutol resistance in clinical *Mycobacterium tuberculosis* isolates. Antimicrob Agents Chemother 2006;50:1900–2.

[36] Ramaswamy S V, Amin AG, Goksel S, Stager CE, Dou S-J, El Sahly H, et al. Molecular genetic analysis of nucleotide polymorphisms associated with ethambutol resistance in human isolates of *Mycobacterium tuberculosis*. Antimicrob Agents Chemother 2000;44:326–36.
