# Supplementary material for: Deep amplicon sequencing for culture-free prediction of susceptibility or resistance to 13 anti-tuberculous drugs
Source: Eur Respir J. 2021 Mar 18;57(3):2002338. doi: 10.1183/13993003.02338-2020 (PMC8174722; doi:10.1183/13993003.02338-2020)
Supplement: Supplementary file 3 [file ERJ-02338-2020.Figure_1.pdf]

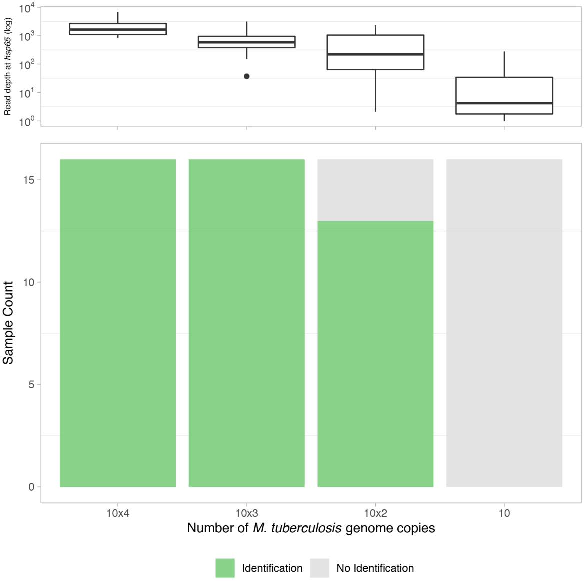

Supplementary Figure 1. Limit of detection (LOD) of Deeplex Myc-TB for mycobacterial identification. (Top) Read depth at *hsp65* versus the number of input genomes. Median values as well as 25-75% quartiles are shown. (Bottom) For each dilution level with 10, 10^2, 10^3, 10^4 genome copies, LOD was measured as the fraction with (green) or without (grey) correct identification among 16 tests, corresponding to four independent amplification replicates and sequencing rounds of 4 MTBC genomic DNA extracts.
