# Supplementary material for: Deep amplicon sequencing for culture-free prediction of susceptibility or resistance to 13 anti-tuberculous drugs
Source: Eur Respir J. 2021 Mar 18;57(3):2002338. doi: 10.1183/13993003.02338-2020 (PMC8174722; doi:10.1183/13993003.02338-2020)
Supplement: Supplementary file 4 [file ERJ-02338-2020.Figure_2.pdf]

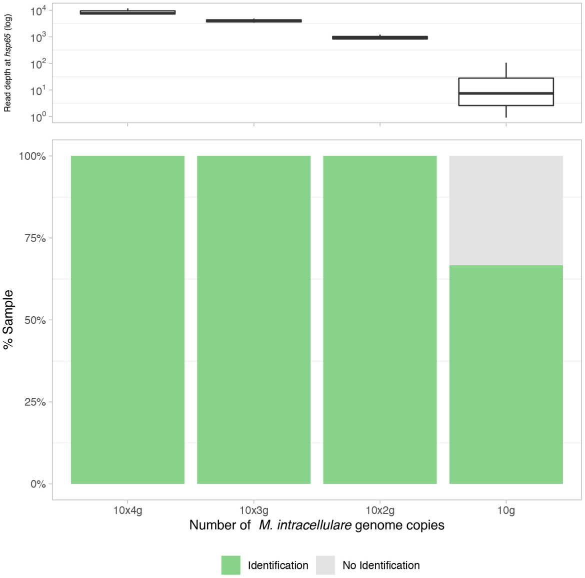

Supplementary Figure S2. Limit of detection (LOD) of Deeplex Myc-TB for NTM identification. (Top) Read depth at hsp65 versus the number of input genomes of a *M. intracellulare* strain. Median values as well as 25-75% quartiles are shown. (Bottom) LOD was measured as the fraction with (green) or without (grey) correct identification in three independent replicated analyses of 10, 10^2, 10^3, 10^4 *M. intracellulare* genome copies.
