# Supplementary material for: Deep amplicon sequencing for culture-free prediction of susceptibility or resistance to 13 anti-tuberculous drugs
Source: Eur Respir J. 2021 Mar 18;57(3):2002338. doi: 10.1183/13993003.02338-2020 (PMC8174722; doi:10.1183/13993003.02338-2020)
Supplement: Supplementary file 6 [file ERJ-02338-2020.Figure_4.pdf]

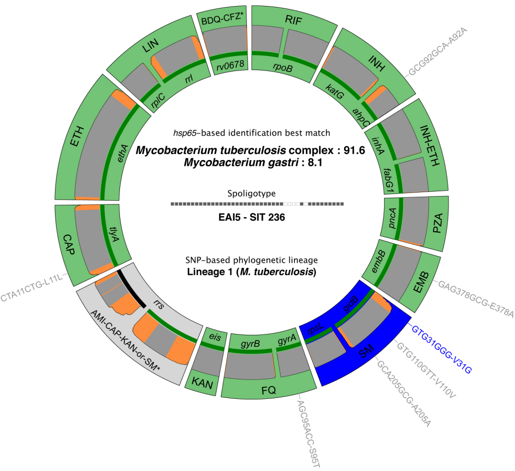

Supplementary Figure S4. Deeplex Myc-TB detecting co-existing *hsp65* reads of *Mycobacterium tuberculosis* complex and *Mycobacterium gastri* strains in DNA from an individual sputum sample collected in a TB drug resistance survey conducted in Djibouti. The respective percentages of *hsp65* reads detected for each species are shown under "*hsp65*-based identification best-match". Explanations in the legend of Figure 2 in the main text apply for other parts of this figure.
