# Supplementary material for: Deep amplicon sequencing for culture-free prediction of susceptibility or resistance to 13 anti-tuberculous drugs
Source: Eur Respir J. 2021 Mar 18;57(3):2002338. doi: 10.1183/13993003.02338-2020 (PMC8174722; doi:10.1183/13993003.02338-2020)
Supplement: Supplementary file 7 [file ERJ-02338-2020.Figure_5.pdf]

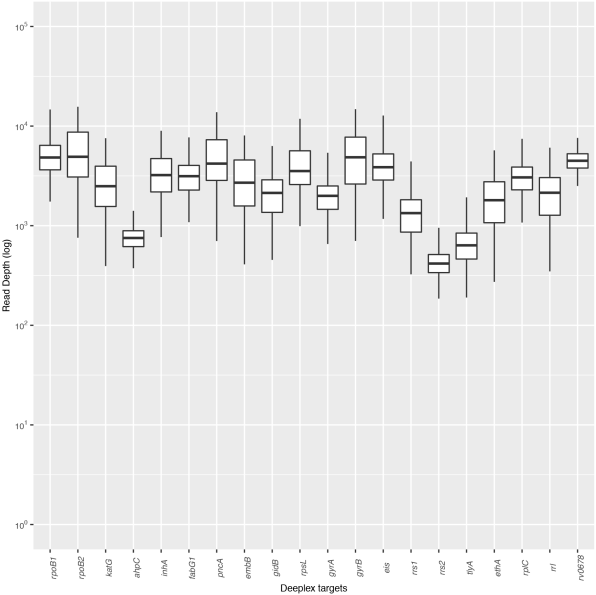

Supplementary Figure S5. Log read depth obtained at drug resistance-associated Deeplex Myc-TB targets in 429 DNA strain samples from the TDR and the Sciensano-Belgian National TB Reference Center collections. Median values as well as 25-75% quartiles are shown.
