# Supplementary material for: Deep amplicon sequencing for culture-free prediction of susceptibility or resistance to 13 anti-tuberculous drugs
Source: Eur Respir J. 2021 Mar 18;57(3):2002338. doi: 10.1183/13993003.02338-2020 (PMC8174722; doi:10.1183/13993003.02338-2020)
Supplement: Supplementary file 8 [file ERJ-02338-2020.Figure_6.pdf]

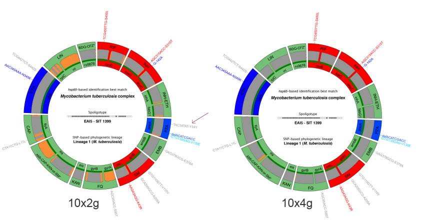

Supplementary Figure S6. Deeplex Myc-TB results obtained using 10^2 versus 10^4 *M. tuberculosis* genome copies of sample TB-TDR0014 from the TDR collection. A red arrow indicates an erratically detected false positive synonymous low frequency variant (5.2%) *pncA* Y34Y at 10^2 genomes. This variant was detected only in this experiment. This likely reflects very sporadic emergence over filtered noise level of low frequency amplification errors, sometimes seen with 10 or 10^2 genome copies.
