# Supplementary material for: Deep amplicon sequencing for culture-free prediction of susceptibility or resistance to 13 anti-tuberculous drugs
Source: Eur Respir J. 2021 Mar 18;57(3):2002338. doi: 10.1183/13993003.02338-2020 (PMC8174722; doi:10.1183/13993003.02338-2020)
Supplement: Supplementary file 9 [file ERJ-02338-2020.Shareable.pdf]

SHAREABLE PDF

Deep amplicon sequencing for culture-free prediction of susceptibility or resistance to 13 anti-tuberculous drugs

Agathe Jouet[1,12], Cyril Gaudin[1,12], Nelly Badalato[1], Caroline Allix-Béguec[1], Stéphanie Duthoy[1], Alice Ferré[1], Maren Diels[2], Yannick Laurent[1], Sandy Contreras[1], Silke Feuerriegel[3,4], Stefan Niemann[3,4], Emmanuel André[5], Michel K. Kaswa[6], Elisa Tagliani[7], Andrea Cabibbe[7], Vanessa Mathys[8], Daniela Cirillo[7], Bouke C. de Jong[9], Leen Rigouts[9,10] and Philip Supply[11]

Affiliations: [1]GenoScreen, Lille, France. [2]BCCM/ITM, Mycobacteria Collection, Institute of Tropical Medicine, Antwerp, Belgium. [3]Molecular and Experimental Mycobacteriology, Research Center Borstel, Borstel, Germany. [4]German Center for Infection Research (DZIF), Partner Site Hamburg–Lübeck–Borstel, Borstel, Germany. [5]Laboratory of Clinical Bacteriology and Mycology, Dept of Microbiology and Immunology, KU Leuven, Leuven, Belgium. [6]National Tuberculosis Program, Kinshasa, Democratic Republic of the Congo. [7]Emerging Bacterial Pathogens, IRCCS San Raffaele Scientific Institute, Milan, Italy. [8]Unit Bacterial Diseases Service, Infectious Diseases in Humans, Sciensano, Brussels, Belgium. [9]Mycobacteriology Unit, Dept of Biomedical Sciences, Institute of Tropical Medicine, Antwerp, Belgium. [10]Dept of Biomedical Sciences, Antwerp University, Antwerp, Belgium. [11]Université de Lille, CNRS, INSERM, CHU Lille, Institut Pasteur de Lille, U1019–UMR 8204–CIIL (Center for Infection and Immunity of Lille), Lille, France. [12]These authors contributed equally to this work.

Correspondence: Philip Supply, Institut Pasteur de Lille, U1019–UMR 8204–CIIL (Center for Infection and Immunity of Lille), 1 Rue du Prof Calmette, 59000 Lille, France. E-mail: philip.supply@ibl.cnrs.fr

@ERSpublications
The novel Deeplex Myc-TB molecular assay shows a high degree of accuracy for extensive prediction of susceptibility and resistance to 13 anti-tuberculous drugs, directly achievable without culture, which may enable fast, tailored tuberculosis treatment https://bit.ly/3bAvcAt

Cite this article as: Jouet A, Gaudin C, Badalato N, *et al.* Deep amplicon sequencing for culture-free prediction of susceptibility or resistance to 13 anti-tuberculous drugs. *Eur Respir J* 2021; 57: 2002338 [https://doi.org/10.1183/13993003.02338-2020].

This single-page version can be shared freely online.

ABSTRACT Conventional molecular tests for detecting *Mycobacterium tuberculosis* complex (MTBC) drug resistance on clinical samples cover a limited set of mutations. Whole-genome sequencing (WGS) typically requires culture.

Here, we evaluated the Deeplex Myc-TB targeted deep-sequencing assay for prediction of resistance to 13 anti-tuberculous drugs/drug classes, directly applicable on sputum.

With MTBC DNA tests, the limit of detection was 100–1000 genome copies for fixed resistance mutations. Deeplex Myc-TB captured *in silico* 97.1–99.3% of resistance phenotypes correctly predicted by WGS from 3651 MTBC genomes. On 429 isolates, the assay predicted 92.2% of 2369 first- and second-line phenotypes, with a sensitivity of 95.3% and a specificity of 97.4%. 56 out of 69 (81.2%) residual discrepancies with phenotypic results involved pyrazinamide, ethambutol and ethionamide, and low-level rifampicin or isoniazid resistance mutations, all notoriously prone to phenotypic testing variability. Only two out of 91 (2.2%) resistance phenotypes undetected by Deeplex Myc-TB had known resistance-associated mutations by WGS analysis outside Deeplex Myc-TB targets. Phenotype predictions from Deeplex Myc-TB analysis directly on 109 sputa from a Djibouti survey matched those of MTBSeq/PhyResSE/Mykrobe, fed with WGS data from subsequent cultures, with a sensitivity of 93.5/98.5/93.1%

Copyright ©ERS 2021. This version is distributed under the terms of the Creative Commons Attribution Licence 4.0.

and a specificity of 98.5/97.2/95.3%, respectively. Most residual discordances involved gene deletions/indels and 3–12% heteroresistant calls undetected by WGS analysis or natural pyrazinamide resistance of globally rare "*Mycobacterium canettii*" strains then unreported by Deeplex Myc-TB. On 1494 arduous sputa from a Democratic Republic of the Congo survey, 14 902 out of 19 422 (76.7%) possible susceptible or resistance phenotypes could be predicted culture-free.

Deeplex Myc-TB may enable fast, tailored tuberculosis treatment.
