# Supplementary material for: Deep amplicon sequencing for culture-free prediction of susceptibility or resistance to 13 anti-tuberculous drugs
Source: Eur Respir J. 2021 Mar 18;57(3):2002338. doi: 10.1183/13993003.02338-2020 (PMC8174722; doi:10.1183/13993003.02338-2020)
Supplement: Supplementary file 5 [file ERJ-02338-2020.Figure_3.pdf]

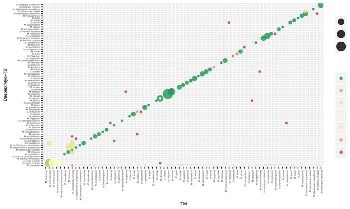

Supplementary Figure 3. Identification of nontuberculous mycobacterial species by Deeplex *Myc*-TB versus reference identification. Deeplex *Myc*-TB results were based on *hsp65* best match analysis, complemented by specific SNP detection in 16S (*rrs*) and 23S rDNA (*rrl*) targets for *M. kansasii* and *M. chelonae* strains, while reference identification was based on *rpoB* and/or 16S rDNA Sanger sequencing results, phenotypic profiling and/or type strain status. The number of isolates studied per taxon (complex, species or subspecies) is proportional to associated circle sizes. FM-A, FM-C: Full match at same taxonomic level (complex, species or subspecies), FM-B: Full match at (sub)species level but with a clonal mix identified by Deeplex, M-D: Match at complex level, sublevel provided by Deeplex, M-E: Match at complex level, sublevel provided by the reference, M-F: Match at complex level, different subspecies, PM: Partial match, several possible species identified by Deeplex, NM: No match. Nontuberculous mycobacterial taxa are ordered phylogenetically, according to Tortoli *et al.* 2019.
